# Breast Cancer Upstaging Risk and In Vivo Tumor Growth Rates Associated with Preoperative Delays

**DOI:** 10.1245/s10434-025-17867-9

**Published:** 2025-07-23

**Authors:** Richard J. Bleicher, Karen J. Ruth, Austin D. Williams, Eric A. Ross, Andrea S. Porpiglia, Allison A. Aggon, Dennis R. Holmes

**Affiliations:** 1 Division of Breast Surgery, Fox Chase Cancer Center, Philadelphia, PA USA; 2 Department of Biostatistics, Fox Chase Cancer Center, Philadelphia, PA USA; 3 Sam and Grace Comprehensive Breast Center, Adventist Health, Glendale, CA USA

**Keywords:** Breast cancer, Delays, Timeliness, Growth rates, Upstaging

## Abstract

**Background:**

Preoperative delay when treating breast cancer confers poorer outcomes, but growth rates and the upstaging likelihoods per delay interval remain unknown. This study evaluated upstaging risk, nodal spread, and tumor growth rates in vivo while awaiting treatment.

**Patients and Methods:**

Registry-based data from the national cancer database was reviewed for patients treated between 2010 and 2020 at commission on cancer-accredited facilities, with nonmetastatic, noninflammatory breast cancer, undergoing surgery first.

**Results:**

Among 1,018,219 patients, 11.5% had primary tumoral upstaging and 14.1% had nodal upstaging. For every 30 d between diagnosis and surgery, the adjusted odds ratios (ORs) for tumor upstaging was 1.11 for ductal carcinoma in situ (DCIS) (95% CI 1.09–1.13, *P *< 0.0001), 1.13 for cT1 (95% CI 1.11–1.15, *P* < 0.0001), and 1.18 for cT2 tumors (95% CI 1.15–1.21, *P* < 0.0001). For invasive tumors, the adjusted 30-d ORs for upstaging in triple negative (TN) primaries were higher (*P* < 0.0001) at 1.21 (95% CI 1.17–1.25) than hormone receptor-positive (HR+, 1.13; 95% CI 1.12–1.15) and human epidermal growth factor 2-positive (HER2+, 1.09; 95% CI 1.04–1.13). cN0 patients had an adjusted OR for upstaging to node-positive of 1.07 (95% CI 1.06–1.08, *P* < 0.0001). The number of 30-d intervals for cT1a, cT1b, cT1c, and cT2 tumors to grow 1 mm was 3.95, 2.63, 2.27, and 1.92, respectively, with tumor growth faster in TN tumors (1.29) than HER2+ (4.95) or HR+ (2.35) (*P* < 0.0001).

**Conclusions:**

Longer delays risk greater upstaging and nodal spread, explaining the association with higher disease-specific and overall mortality in prior studies. Larger and TN tumors have larger delay-associated upstaging likelihoods and in vivo growth rates, making preoperative delays more impactful in these groups.

**Supplementary Information:**

The online version contains supplementary material available at 10.1245/s10434-025-17867-9.

Breast cancer accounted for 31% of all diagnosed cancers and 15% of all cancer-related deaths in 2023.^1^ Survival for the disease has improved, but times between diagnosis and treatment have been increasing over the past two decades.^2^ The reasons for longer delays are myriad, but we have found that necessary components of the workup, such as imaging and biopsies,^2^ desirable factors such as multidisciplinary preoperative evaluation,^3^ and patient behaviors out of physician control, such as transfers of care and second opinions,^4^ all contribute to surgical delays.

It has also been established that longer times between breast cancer diagnosis and surgery, in the non-neoadjuvant setting, contribute to poorer overall and disease-specific survival,^5^ regardless of phenotype.^6^ For every 30-d increment there is a 9–10% relative drop in overall survival, and for each 60-d interval there is a relative 26% increase in breast-cancer specific mortality.^5^ Only recently did the Commission on Cancer establish a breast cancer quality measure to collect data on the numbers of surgical procedures occurring ≤ 60 d of diagnosis.^7^ Although survival is the most important oncologic outcome, statistics enumerating the relative increase in mortality can be challenging for patients and physicians to interpret.^8^

Breast cancer stage is a concept familiar to patients and physicians alike and provides information about extent of disease, treatment options, and mortality risk from a known tumor burden. In order to gauge a patient’s prognosis, stage groupings provide the most robust survival estimation. In our experience, patients are uniquely focused on their tumor stage, and likelihood of upstaging while awaiting treatment. They have particular concern for their risk of preoperatively converting from clinically node-negative to pathologically node-positive. It remains unknown, however, what length of delay is associated with these changes and there is no national data, to our knowledge, about tumor growth rates in vivo while patients await treatment.

Typically, to determine growth rates, one would look to tumor doubling times (i.e., cell division time). Unfortunately, such investigations have been highly variable and of little use, with estimates ranging from 3 d to 19.3 years.^9,10^ This study was undertaken to determine primary tumor and nodal upstaging likelihoods associated with preoperative delay intervals, and to estimate tumor growth rates in vivo while awaiting treatment.

## Patients and Methods

### Patients

After American College of Surgeons’ National Cancer Database (NCDB) and Fox Chase Cancer Center Institutional Review Board approvals, NCDB records were reviewed for patients with nonmetastatic breast cancer diagnosed between 2010 and 2020. The NCDB is the largest national United States dataset to contain the clinical and pathologic American Joint Committee on Cancer (AJCC) staging required for this study, generalizable to the population at large for most breast cancer investigations.^11^ A prospective study is infeasible because of the ethics of subjecting patients to delay, and sample sizes required for sufficient power to investigate this topic.

We identified patients with biopsy-proven, staged breast cancer who had surgery as first treatment. Patients were excluded who had neoadjuvant therapy, surgery > 180 d after diagnosis, or if this was not their first malignancy.

**Fig. 1:**
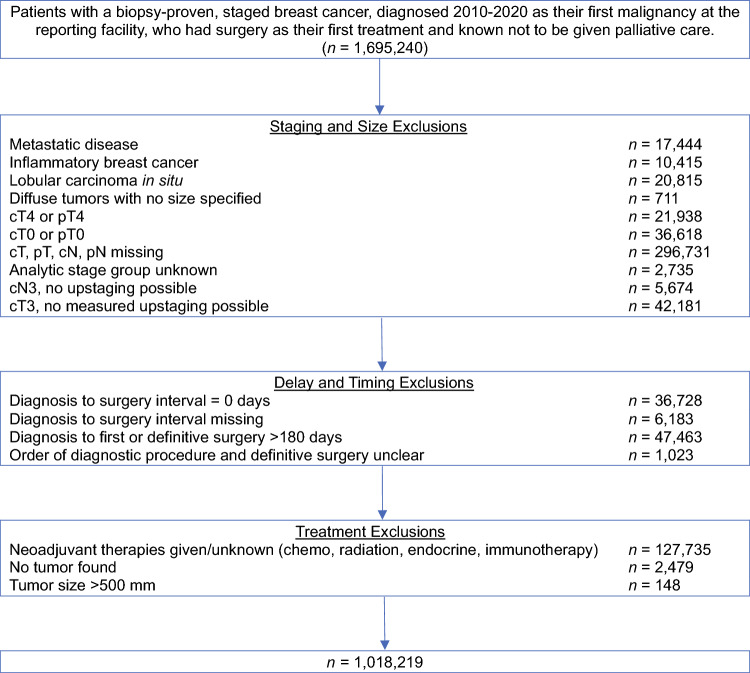
STROBE diagram of cohort inclusions and exclusions

Exclusions are enumerated in Fig. 1. Complete method details are elaborated in the Supplementary Methods and Supplementary Table 1.

### Delay and Upstaging Definitions

The term “delay” here refers to any time interval from date of diagnosis to the date of the first or only surgery, irrespective of clinical impact. These intervals are described here in days or 30-day intervals.

Breast tumor upstaging was defined as any increase from clinical T stage to surgical pathologic T stage, categorized as Tis, T1, T2, and T3. Similarly, nodal upstaging was any increase from clinical to surgical pathologic stage, with N-stage categorized as N0, N1, N2, and N3.

Clinical stage is not completely accurate,^12^ and a patient’s pathologic upstaging can reflect clinical understaging due to the inaccuracies of physical examination or imaging. Pathologic upstaging may also be due to tumor growth or disease that has spread to lymph nodes during the interval between clinical and pathologic staging. To determine the upstaging specifically attributable to delay-related growth and disease advancement, total upstaging rates were adjusted by subtracting estimated rates of clinical staging inaccuracy at diagnosis. Those inaccuracy rates were determined from patients whose surgery was ≤ 15 d from diagnosis, assuming negligible growth in that short interval. Consequently, the upstaging rate due to delay is the rate of upstaging overall, less the rate of inaccuracy at diagnosis. Because downstaging is less of a concern, it was included in the group that was not upstaged. In separate analyses, we examined tumor and nodal upstaging as a binary variable (present or not), and by the degree of upstaging (i.e., one-stage versus  ≥  two-stage increase).

### Statistical Analysis for Upstaging

Our primary hypothesis was that longer delays are associated with growth, demonstrable by an increase in the likelihood of T- and N-stage upstaging. We used multivariable logistic regression to obtain average adjusted predicted probabilities of upstaging for increasing delay intervals with facility cluster-adjusted robust standard errors. We initially examined the diagnosis-to-surgery intervals as categorical and continuous variables. For T upstaging, we ran separate models for each clinical T stage (any Tis, cT1, and cT2), with covariates selected *a priori* including age, sex, race, Hispanic ethnicity, phenotype, grade, histology, and cN stage. In a separate model for nodal upstaging, we included cN0 patients, where the outcome was upstaging to nodal positivity (pN1, pN2, or pN3), which is more clinically impactful than upstaging from cN1 or cN2. The logistic model results were presented as adjusted predicted probabilities of upstaging for delay (continuous) at 15 d (“staging inaccuracy” interval) and 30-d intervals, and the corresponding odds ratio for a difference of 30 d.

For delay intervals of 30, 60, …180 d, we calculated the *delay-attributable probability* of upstaging by subtracting the probability of inaccurate upstaging from the estimate of upstaging at that delay interval.

Models assessing phenotype excluded ductal carcinoma in situ (DCIS) and tumors of unknown phenotypes. Odds ratio (OR) estimates for phenotypes were determined from separate logistic regression models, each adjusting for age, sex, race, Hispanic ethnicity, grade, histology, and cT and cN stages.

### Statistical Analysis for Growth Rates

We inferred growth rates of tumors from analyses of the regression of pathologic tumor size on delay interval (Supplementary Methods). The association of tumor size (mm) and delay (days) was examined in an analytic subgroup of 711,593 patients having measurable invasive cancer, whose delays were beyond the baseline inaccuracy interval (> 15 d) and did not have missing tumor size or phenotype. For these analyses, we included patients with cT1a, cT1b, cT1c, cT1-not-otherwise-specified (NOS), and cT2 tumors, as these subcategories reflect the initial tumor size. Associations between tumor size and delay were examined using multivariable linear regression, with the slope of tumor size on delay providing an estimate of tumor growth (e.g., mm per 30-d interval). The reciprocal of this growth rate estimated the delay interval associated with 1 mm of growth (i.e., expressed as d/mm).

For such large cohorts, statistical significance should not be the basis for interpreting the results. All tests were two-sided with 1% type I error instead of 5%, given the large sample size, but clinical significance was still the primary consideration for all outcomes. Analyses were conducted using SAS 9.4 (SAS Institute, Cary NC) and Stata 15 (StataCorp, College Station, TX), and figures were created using SAS, Stata, and Excel.

## Results

There were 1,018,219 patients after exclusions (Fig. 1). Overall, median age was 62 years old (interquartile range [IQR] 52–70 years), < 1% were men, 83.2% white, 10.4% Black, 4.2% Asian, and 2.1% other/unknown, with 5.5% reporting Hispanic ethnicity throughout. Cohort characteristics are enumerated by clinical T stage in Supplementary Table 2 and clinical N stage in Supplementary Table 3. Overall, mean and median delays between diagnosis and surgery were 38.5 ± 22.2 d (mean ± standard deviation) and 34 [23–49] d (median [IQR]), with 10.1% of patients having a time to surgery ≤ 15 d. The distribution of delays overall and stratified by the presence or absence of T-stage upstaging are shown in Supplementary Figs. 1 and 2.

### Upstaging

Among all patients (including those in the initial 15-d window), 11.5% had their breast tumor upstaged, but this differed by clinical T stage, with upstaging proportions of 15.8%, 12.1%, and 5.3% for cTis, cT1, and cT2, respectively (*P* < 0.0001; Supplementary Fig. 3). Relatively fewer patients were upstaged as the tumor stage category (and growth needed to achieve the next category) increased. In cTis, cT1, and cT2 patients, staging inaccuracy proportions based on the initial 15-d window (i.e., understaging) were 13.9%, 11.4%, and 4.6% (*P* < 0.0001), respectively. In those with delays of 16–180 d, upstaging proportions were higher, with 15.9%, 12.2%, and 5.4% for cTis, cT1, and cT2, respectively (*P* < 0.0001). Supplementary Fig. 4 shows estimated covariate-adjusted upstaging probability curves from logistic regression models (delay as a continuous variable). Upstaging due to preoperative surgical delay (“delay-attributable”) for each interval was estimated by subtracting staging inaccuracy from the interval probability and is enumerated in Table 1 by interval, and stratified by lobular versus ductal histology in Supplementary Table 4. From these logistic models for tumor upstaging for cTis, cT1, and cT2, the adjusted ORs for each 30 d of preoperative delay were 1.11 for cTis (95% CI 1.09–1.13, *P* < 0.0001), 1.13 for cT1 (95% CI 1.11–1.15, *P* < 0.0001), and 1.18 for cT2 (95% CI 1.15–1.21, *P* < 0.0001).

**Table 1:** The adjusted probabilities of primary tumor upstaging by clinical T stage and by nodal upstaging for cN0 patients, for each 30-d delay increment between diagnosis and surgery

Delay interval	cTis (cN0 only) adjusted probability of upstaging (%)			cT1* adjusted probability of upstaging (%)			cT2 adjusted probability of upstaging (%)			cN0 adjusted probability of upstaging (%)		
	Est.	95% CI	Delay-attributable	Est.	95% CI	Delay-attributable	Est.	95% CI	Delay-attributable	Est.	95% CI	Delay-attributable
Staging inaccuracy^#^	14.5	(14.1–15.0)	0	11.2	(10.9–11.5)	0	4.7	(4.5–4.9)	0	13.6	(13.4–13.8)	0
30 d	15.1	(14.7–15.6)	0.6	11.8	(11.5–12.1)	0.6	5.0	(4.9–5.2)	0.4	13.9	(13.8–14.1)	0.4
60 d	16.4	(15.9–16.8)	1.8	13.1	(12.8–13.4)	1.9	5.9	(5.7–6.1)	1.2	14.7	(14.5–14.9)	1.1
90 d	17.7	(17.1–18.3)	3.1	14.5	(14.0–14.9)	3.3	6.8	(6.5–7.1)	2.1	15.4	(15.2–15.7)	1.9
120 d	19.1	(18.3–19.9)	4.5	16.0	(15.3–16.6)	4.8	7.9	(7.3–8.4)	3.2	16.2	(15.8–16.6)	2.7
150 d	20.5	(19.5–21.6)	6.0	17.6	(16.7–18.5)	6.4	9.1	(8.2–9.9)	4.4	17.0	(16.5–17.6)	3.5
180 d	22.1	(20.7–23.5)	7.5	19.3	(18.1–20.6)	8.1	10.4	(9.3–11.6)	5.8	17.9	(17.2–18.6)	4.3

Odds ratio for delay (difference of 30 d):	T-stage upstaging									Nodal upstaging		
	cTis (cN0 only, *n* = 187,734)			cT1 (*n* = 641,651)			cT2 (*n* = 188,718)			cN0 (*n* = 970,778)		
	OR	95% CI		OR	95% CI		OR	95% CI		OR	95% CI	
	1.11	1.09–1.13	*P* < 0.0001	1.13	1.11–1.15	*P* < 0.0001	1.18	1.15–1.21	*P* < 0.0001	1.07	1.06–1.08	*P* < 0.0001

Results of four separate logistic regression models, one for each cT group and also for cN0. The outcome is upstaging (yes/no), with delay from diagnosis to surgery included as a continuous variable (30-d intervals). Covariates included age, gender, race, Hispanicity, pathologic grade, histology, and phenotype. For cTis, the cohort is restricted to cN0, and phenotype adjustment was based on ER and PR receptors as HER2 status was not typically determined. For cT1 and cT2, cN stage is included as a covariate. Some calculations may not add precisely due to rounding

^*^cT1 includes cT1a, cT1b, cT1c, and cT1 not otherwise specified (NOS) all combined. Age is a linear continuous variable (in the logistic model), gender defined as male versus female, and race was categorized as white, Black, Asian, and other/unknown. Grade was categorized into four groups: grade 1 or well-differentiated, grade 2 or moderately differentiated, grade 3 or poorly differentiated or undifferentiated, and grade unknown or missing. Histology was grouped into ductal, lobular, and other/unknown. Lobular carcinoma in situ was excluded from the Tis stage grouping. The phenotype covariate was categorized as hormone receptor-positive (HR+), human epidermal growth factor 2-positive (HER2+), triple negative (TN), and unknown/missing. *Est.* estimate

^#^Inaccuracy refers to the baseline understaging rate that occurs without significant delay. This is calculated by determining the rate of upstaging for patients having times between diagnosis and surgery ≤ 15 d, assuming negligible growth within that time period. The delay-attributable portion is calculated by subtracting this staging inaccuracy from the upstaging rate associated with significant delay, to achieve the upstaging rate attributable to delay

For lymph nodes, 14.6% were upstaged overall. Among all cN0 patients upstaged to clinically node-positive (cN+), 90.1% had delays of 16–180 d versus 9.9% when time to surgery was 1–15 d. For cN0 patients, proportions of those upstaging ≤ 15 and > 15 d were 14.2% and 14.1% respectively, but these are unadjusted. Meanwhile, their 60-d delay probability of nodal upstaging was 14.7%, with 1.1% delay-attributable; at 180 d, the interval and delay-attributable probabilities were 17.9% and 4.3%. The adjusted OR for each 30-d delay was 1.07 (95% CI 1.06–1.08, *P* < 0.0001; Table 1 and Supplementary Fig. 4).

For tumor upstaging by phenotype, adjusted predicted probabilities by delay were higher for TN as compared with HR+ and HER2+ (Table 2 and Supplementary Fig. 4). For TN patients, the 60-d adjusted probability was 13.1%, with 2.8% delay-attributable; 180-d adjusted probabilities were 23.7% and 13.4% delay attributable. For HR+, HER2+, and TN tumors, the adjusted 30-d ORs for upstaging were 1.13 (95% CI 1.12–1.15, *P* < 0.0001), 1.09 (95% CI 1.04–1.13, *P* < 0.0001), and 1.21 (95% CI 1.17–1.25, *P* < 0.0001), respectively. When stratifying by histology, delay-attributable upstaging for lobular, HER2+, and TN tumors did not reach significance (Supplementary Table 5).

**Table 2:** The adjusted probabilities of primary tumor upstaging by phenotype, for each 30-d delay increment between diagnosis and surgery

Delay	HR+			HER2+			TN		
	Estimate	95% CI	Delay-attributable^#^	Estimate	95% CI	Delay-attributable^#^	Estimate	95% CI	Delay-attributable^#^
Staging inaccuracy^#^	9.7	(9.5–10.0)	0	9.8	(9.4–10.3)	0	10.3	(9.9–10.7)	0
30 d	10.3	(10.0–10.6)	0.5	10.2	(9.8–10.6)	0.4	11.1	(10.8–11.5)	0.9
60 d	11.5	(11.2–11.7)	1.7	11.0	(10.5–11.4)	1.1	13.1	(12.6–13.6)	2.8
90 d	12.7	(12.3–13.1)	3.0	11.8	(10.9–12.6)	1.9	15.3	(14.4–16.2)	5.0
120 d	14.1	(13.5–14.7)	4.4	12.6	(11.3–13.9)	2.7	17.8	(16.4–19.3)	7.6
150 d	15.6	(14.8–16.4)	5.9	13.5	(11.6–15.3)	3.6	20.6	(18.5–22.7)	10.3
180 d	17.3	(16.2–18.3)	7.5	14.4	(12.0–16.9)	4.6	23.7	(20.8–26.6)	13.4

Odds ratio for delay (difference of 30 d):	T stage upstaging								
	HR+ (*n* = 681,653)			HER2+ (*n* = 54,632)			TN (*n* = 67,601)		
	OR	95% CI		OR	95% CI		OR	95% CI	
	1.13	1.12–1.15	*P* < 0.0001	1.09	1.04–1.13	*P* < 0.0001	1.21	1.17–1.25	*P* < 0.0001

Results of three separate logistic regression models, one for each invasive cancer phenotype group (hormone receptor-positive [HR+], human epidermal growth factor 2-positive [HER2+], and triple negative [TN]). Phenotypes that were not discernible due to missing data were excluded. The outcome is upstaging (yes/no), with the delay between diagnosis and surgery included as a continuous variable (30-d intervals). Covariates included age, gender, race, Hispanicity, pathologic grade, histology, cT stage (cT1 or cT2), and cN stage. Some calculations may not add precisely due to rounding

cT = Tis excluded, as phenotype is for invasive primary tumors. Age is a linear continuous variable (in the logistic model), gender defined as male versus female, and race was categorized as white, Black, Asian, and other/unknown. Grade was categorized into four groups: grade 1 or well differentiated, grade 2 or moderately differentiated, grade 3 or poorly differentiated or undifferentiated, and grade unknown or missing. Histology was grouped into ductal, lobular, and other/unknown

^#^Inaccuracy refers to the baseline understaging rate that occurs without significant delay. This is calculated by determining the rate of upstaging for patients having times between diagnosis and surgery ≤ 15 d, assuming negligible growth within that time period. The delay-attributable portion is calculated by subtracting this baseline inaccuracy from the upstaging rate associated with significant delay, to achieve the upstaging rate attributable due to delay

### Tumor Growth Rates

**Fig. 2:**
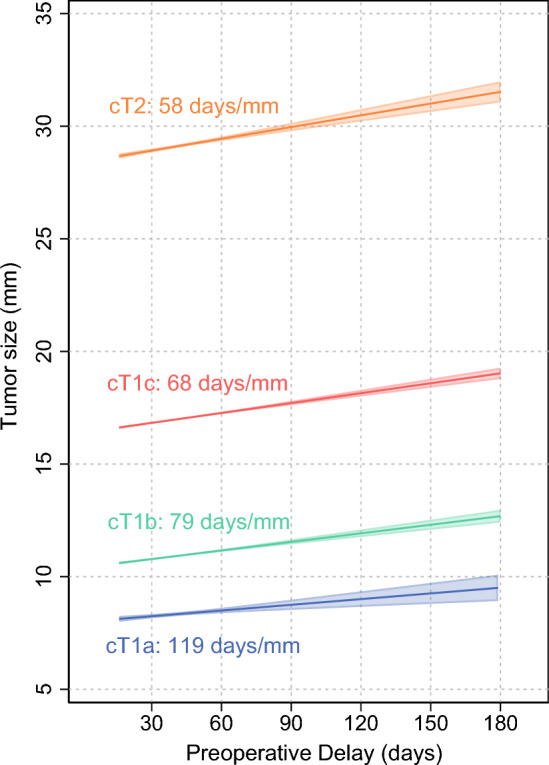
Regression curves demonstrating the rate of growth by T stage: the slopes of growth rates from regression analysis in vivo are depicted, demonstrating the increasing rate of growth by tumor size/substage; in addition, Supplementary Fig. 5 shows these regression curves concatenated and juxtaposed to demonstrate the theoretical increase in growth rates as tumors enlarge

An interaction term for delay and clinical T stage (cT1a, cT1b, cT1c, cT1 NOS, and cT2) to determine whether the slope of tumor size on delay differed by clinical T stage was significant (*P* = 0.005). The regression coefficients (tumor growth) increased from 0.25 mm/30-d for cT1a to 0.52 mm/30-d for cT2. The interval for 1 mm of growth for cT1a tumors was 119 d (95% CI 80–232 d), and 58 d (95% CI 48–71 d) for cT2 (Fig. 2). These curves, when concatenated (Supplementary Fig. 5) demonstrate progressively increasing rates with enlargement. Growth rates for cT1 substages are enumerated in Table 3.

**Table 3:** Linear regression results for tumor size (mm) on delay (30-d intervals) by clinical T substage

Stage	*n*	Coefficient for tumor size on delay: mm/30-d interval	95% CI	*P* value for pairwise difference from cT2 (see note 2)	Number of 30-d intervals for 1 mm of tumor growth
cT1a	49,673	0.253	0.129–0.377	0.0007	3.95
cT1b	163,819	0.381	0.322–0.440	0.0093	2.63
cT1c	244,936	0.440	0.387–0.492	0.0896	2.27
cT1 NOS	90,533	0.388	0.290–0.486	0.0386	2.58
cT2	162,632	0.521	0.422–0.621	Referent	1.92

To assess whether the slope of tumor size on delay differed by cT stage, a separate model was run with all stages combined and with an interaction term for cT stage and delay (continuous), which was statistically significant, *P* = 0.005. Models included patients delayed > 15 d (i.e., delays from 16 to 180 d). The number of 30-d intervals required for 1 mm of tumor growth is the reciprocal of the slope

NOS, not otherwise specified; CI, confidence interval

For growth rates by phenotype (Table 4), the interaction *P*-value for delay was significant (*P* < 0.0001). In a model adjusting for clinical T stage, the 30-d regression coefficient for HR+ tumors (*N* = 608,924) was 0.43 mm (95% CI 0.39–0.47; i.e., 0.43 mm tumor growth per 30-d interval), HER2+ (*N* = 44,523) was 0.20 (95% CI 0.06–0.34), and TN (*N* = 58,146) was 0.78 (95% CI 0.64–0.91). Compared with HR+, the growth rate for HER2+ tumors was lower (*P* = 0.0026), and TN higher (*P* < 0.0001), although HER2+ tumor growth rates were not affected by their HR status (interaction *P* = 0.5). Coefficient reciprocals (days for 1 mm growth) were 71 d for HR+, 148 d for HER2+, and 39 d for TN.

**Table 4:** Linear regression results for tumor size (mm) on delay (30 d intervals) by phenotype, with adjustment for clinical T stage. The phenotype results above are from one linear regression model for tumor size (mm) on delay (30-d intervals)

Phenotype	*n*	Coefficient for tumor size on delay: mm/30-d interval	95% CI	*P* value for pairwise difference from ER+	Number of 30-d intervals for 1 mm of tumor growth
ER+/PR+	608,924	0.426	0.387–0.465	Referent	2.35
HER2+	44,523	0.202	0.062–0.343	0.0026	4.95
TN	58,146	0.775	0.643–0.906	< 0.0001	1.29

The model included clinical T stage (cT1a, cT1b, cT1c, cT1 NOS, or cT2), delay (in continuous fashion), phenotype (HR+, HER2+, TN), and interaction between delay and phenotype. The interaction term for phenotype and delay was statistically significant, P < 0.0001. To further investigate the HER2+ phenotype’s growth rate, patients were then subdivided into HR+/HER2+ and HR−/HER2+. Within HER2+, growth rates based upon HR status did not differ (interaction *P* = 0.5). Growth rates, expressed as number of 30-d intervals for 1 mm of tumor growth were 5.78 for HR+/HER2+ and 3.60 for HR−/HER2+. The coefficient (mm/30-d interval) for HR+/HER2+ was 0.173 (95% CI − 0.011 to 0.356) and for HR−/HER2+ was 0.278 (95% CI 0.023 to 0.532)

NOS, not otherwise specified; CI, confidence interval; ER, estrogen receptor; PR, progesterone receptor; HER2, human epidermal growth factor 2; TN, triple negative

## Discussion

Although we often think of healthcare delays as a modern concern, even William Halsted opined in 1907 that “the slightest delay [in breast cancer treatment] is dangerous…”^13^ because delay may predispose to stage advancement, which can become incurable. Although delays have long existed,^14^ population-based series evaluating their impact have occurred primarily within the past two decades.^5,15^ These have also demonstrated recent increases in their magnitude,^2^ with disparities that still exist.^16^ Treatment delays are problematic because of their effect on outcomes, but they also result in significant patient anxiety.

In our experience, the single most expressed concern in patient consultations and pretreatment visits is whether time to treatment, and surgery in particular, will allow an opportunity for the cancer to grow, upstage, and spread to lymph nodes or distant sites, if it has not already. Patients focus on stage, and whether pretreatment delays will result in larger tumors, by inquiring how fast breast cancers grow before they are excised and treated. In those who are clinically node-negative, the focus is frequently on whether they will become node-positive at surgery from waiting for treatment.

When considering the effects of delays on patients, survival remains most important, but treatment morbidities often also increase with stage. We have previously found that survival is impaired by longer times to surgery.^5^ While we assume that delays are problematic because they allow tumors time to grow and spread, technically this is an assumption; there has been little prior data supporting this. Although indirect data on growth rates exists, there has also been no comprehensive national data to date estimating tumor growth rates in vivo using pathologic tumor size.

Although the 30-d delay interval odds ratios for upstaging increased from 1.11 for cTis to 1.13 for cT1 and 1.18 for cT2 tumors, the percentages of tumors in those categories that upstaged declined from 15.8% to 12.2% to 5.3%, respectively. This is likely because the range of each progressively larger T stage widens, necessitating more growth for the tumor to advance to the next level. The Tis category merely requires the development of invasion of any size to advance to T1, whereas T1 must outgrow its 2-cm range of 0.1–2 cm to become T2, and T2 must outgrow the 2–5 cm range to become T3. Surely, upstaging also declines as tumors become larger because they are more clinically apparent and accurately sized, and understaging becomes less likely. Our finding that lobular tumors have a higher risk of upstaging than ductal tumors is also consistent with their growth patterns and the challenges lobular tumors present on palpation and imaging. We believe that upstaging may have lacked significance for lobular TN and HER2-positive tumors because of the smaller sample sizes in those subgroups.

One study assessing upstaging by Fischer and colleagues^17^ using the NCDB, evaluated categorical delay intervals of 4 weeks on patients having clinically negative axillary nodes. They found that the likelihood of nodal-positivity was 4–5% per month, with a 5.3% increase in nodal-positivity every 4 weeks. Although they did not assess or adjust for clinical inaccuracy to ensure that the upstaging was due to delay, as we have here, their findings resembled our calculated 7% increase in the odds of upstaging for each 30-d of delay. We adjusted for clinical understaging because the inaccuracies associated with clinical examination and imaging are not trivial, with nodal understaging present here in 13.6% of cases, and primary tumoral understaging in 4.7–14.5% of cases.

We also assessed in vivo growth rates during patient treatment delays, with particular attention paid to established beliefs that cancer growth rates may not be uniform. The predominating theory maintains that tumor growth proceeds in Gompertzian, or serpiginous, fashion^18^, with rates that progressively increase until tumors outgrow their space and blood supply, when their growth rates plateau. Although prior studies predominantly use mammographic estimates for tumor growth, the correlation value for mammographic estimation to pathologic tumor size varies.^19,20^ Mammography is more accurate than examination,^21^ but less accurate than ultrasound and magnetic resonance imaging (MRI).^22,23^ The most accurate measure of growth is pathologic size, and so we created regressions of each clinical tumor stage, for delay versus pathologic tumor size. A large national dataset was needed to achieve sufficient power for these determinations and demonstrated that tumors do increase their growth rates as they enlarge. Consequently, when concatenated (Supplementary Fig. 5), these confirm the Gompertizian or s-shaped growth curve that has been theorized for the past 40 years.^18^ This is helpful as we cannot ethically leave tumors in patients to serially measure them in vivo as they grow in order to confirm such a pattern.

These data suggest that as tumors enlarge, their rates likely increase from more tumor cells dividing at any one point in time. Despite these findings, however, the growth rates were noted to be slow, with the most rapid tumors being the largest evaluated (T2 tumors at 58 d/mm) and TNs (at 39 d/mm). It is worthy of emphasis that these growth rate regressions represent an amalgamation of all tumors, so some will grow faster and some slower. Since tumor growth begins before diagnosis, most of their lifespan is felt to be during the “silent interval,” long before we are capable of knowing they are present.^9^

This also supports the finding that rates of growth are indeed slow. Our own recall bias for the fastest and most concerning tumors likely predisposes us to think that most tumors grow quickly. Even if not completely accurate, prior mammography data does at least suggest that some breast cancers grow so slowly that there is no discernible size increase in diameter over the course of even 2 years.^10^ The upstaging likelihoods seen here are consistent with these slow growth rates, as even with 180-d delays for cTis, cT1, and cT2 tumors, 66%, 58%, and 45% of the total upstaging probabilities were due to clinical staging inaccuracy and not the delay itself. For cN0 tumors, this accounted for a remarkable 76% of the risk at 180-d. With a 3–5% survival threshold accepted by many trials,^24,25^ we believe a ≤ 5% delay-attributable upstaging risk to be tolerable, as some upstaging (e.g., 1.9 cm versus 2.1 cm) is not clinically impactful and should result in a well-below 3% risk to outcomes.

In addition to larger tumors growing faster than smaller ones, TN tumors also grow more rapidly than other phenotypes. TN tumors confer their survival impairment by early metastatic disease and not by local factors, and the TN tumors in this cohort had the highest proportion of high-grade lesions (TN at 76% versus 15% and 53% for HR+ and HER2+, respectively), potentially explaining this finding and their propensity to metastasize. This may be consistent with prior data. One study evaluating tumor doubling times in 12 cancers using a statistical approach from progression-free survival,^26^ found TN breast cancers do grow faster than those that are HR+ and HER2+. Although HER2+ tumors grew slightly faster than HR+, their doubling times overlapped with them. Another retrospective series evaluating 323 ultrasound studies^27^ found that while HR status predicted growth rate, HER2 status did not. Although we found that HER2-positive tumors grew slower, these previously published studies were quite small with indirect methods of assessment. This study, with over 1 million patients, may be able to discern differences that those could not.

We have previously evaluated and published comparative delay outcomes by phenotype,^6^ noting no significant differences between them in the relative survival decline for any given delay interval. One possible explanation for this seeming contradiction between growth and outcomes is that the differences in growth rates seen here may not translate into a delay-related survival decline. Another may be that the relative declines in survival due to delays are not statistically discernible, but the absolute declines are different, meaning that no contradiction exists. Some tumors may also metastasize at smaller sizes while the primary continues to grow, making some delays and tumor size irrelevant.

There are several caveats to this study that should be mentioned. Realistically, analyses assessing survival impairments due to delay need to be retrospective, as ethical considerations prohibit a prospective trial that subjects patients with cancer to varying delays. However, non-level I data does have a risk of unmeasured confounding. One cannot rule out correlates to both the delay and the outcome impairment, despite adjusting for patient factors (such as comorbidities), tumor characteristics, and treatment specifics. A double association could create the appearance of an association where none exists. Also, exclusion of those having neoadjuvant chemotherapy could create a biased sample. The NCDB does not have an estimate of clinical tumor size, which might have improved the specificity of the growth rate regressions. Finally, because we cannot know how any particular tumor was clinically staged, the heterogeneity of clinical staging inaccuracy is also unknown, as is its effect on our delay-attributable upstaging estimates.

Importantly, the data in this study corroborates what has been assumed to be the mechanism by which treatment delays impair outcomes: upstaging, growth, and spread of disease. These data support the association between survival and timely therapy by confirming the suspected mechanism for this association and quantifying the risk of upstaging and nodal spread, as well as growth rates in vivo for a given time interval. These provide needed data for both patients and clinicians. As primary breast cancers having higher T stages and those that are TN have long been known to have poorer survival, their more rapid growth further confirms that timely care should be sought to maximize outcomes. 

## Supplementary Information

Below is the link to the electronic supplementary material.

Supplementary file1 (DOCX 36 KB)

Supplementary file2 (DOCX 101 KB)

Supplementary file3 (DOCX 677 KB)
