# Supplementary material for: Breast Cancer Upstaging Risk and In Vivo Tumor Growth Rates Associated with Preoperative Delays
Source: Ann Surg Oncol. 2025 Jul 23;32(12):8789–97. doi: 10.1245/s10434-025-17867-9 (PMC12534249; doi:10.1245/s10434-025-17867-9)
Supplement: Supplementary file 1 — Supplementary file1 (DOCX 36 KB) [file 10434_2025_17867_MOESM1_ESM.docx]

**SUPPLEMENTARY METHODS**

**Data Source and Sample**

After approval by the American College of Surgeons’ National Cancer Database (NCDB) and Fox Chase Cancer Center Institutional Review Board, patient records in the NCDB 2020 Participant User File (PUF) were reviewed for the inclusion/exclusion criteria (below). The NCDB was chosen for the study because it is the only national dataset to contain nearly uniform clinical and pathologic American Joint Committee on Cancer (AJCC) staging for patients aged 18 and older, required for this study. This dataset also contains approximately 70% of the cancers in the United States treated at Commission on Cancer-accredited centers, and although hospital based, is felt to be generalizable to the population at large for most breast cancer investigations. A prospective study was not considered feasible because of the ethical considerations of subjecting patients to delay, and the sample size required to have sufficient power to investigate this topic.

**Inclusion and Exclusion Criteria**

To examine the association between delay and tumor growth or spread, we included nonmetastatic, non- inflammatory breast cancer patients, diagnosed with their first or only breast cancer between 2010 and 2020 who received surgery without neoadjuvant therapy of any type. Inclusion started with 2010, when complete staging and HER2 information were routinely collected by the NCDB. We excluded tumors recorded as >500 mm, and those having surgery >180 days after diagnosis as we felt there was not adequate data available to support statistical inferences beyond this point. Since the primary outcome of interest was upstaging, we excluded patients with missing data for clinical or pathologic T and N stages. We also excluded patients where staging was not based on tumor size (cT4, pT4), no evidence of tumor (cT0,pT0) and where upstaging based on growth could not be determined (cT3, cN3). The exclusion specifics are enumerated in **Figure 1 and eTable 1**. In accordance with NCDB privacy requirements, cells and groups <10, or cells and groups enabling enumeration of those <10 have been censored.

Patients having their first invasive or *in situ* breast cancer were included, while lobular carcinoma *in situ* was excluded. Patients were excluded if their diagnosis was not made by needle biopsy or excisional biopsy. Since our interest is in growth occurring during the interval from diagnosis to surgery (prior to adjuvant therapy), we excluded patients who did not receive surgery, as well as those receiving neoadjuvant treatment.

For those with missing data for pathologic T stage, tumor size was used to calculate missing values where possible. Similarly, those having a missing pathologic N stage were classified into the appropriate stage using the number of nodes listed as containing metastatic disease. Patients were kept in the cohort if they had complete primary tumor staging and nodal staging information, i.e. both clinical and pathologic.

**Variable Definitions**

**Delay:** The interval in days from diagnosis to first surgical procedure was defined as the delay interval, with the assumption that the bulk of tumor and consequently the pathologic tumor size would have been determined at this surgery (e.g. re-excisions typically do not excise the bulk of tumor). Because the impact of delays is continuous and gradual, and because there is no agreed-upon cut point at which the time interval between diagnosis and surgery suddenly becomes clinically relevant, the term ‘delay’ was used here for any time interval for uniformity, clarity, and space considerations.

**Upstaging:** Our primary objective was to examine the effect of surgical delay on tumor growth or spread, using breast T-upstaging and nodal upstaging as indirect measures of these increases. Breast T stage upstaging was defined as an increase in stage from initial clinical T stage to surgical pathologic T stage that reflected measurable tumor size growth, with T stage categorized as any Tis, T1, T2, and T3. Similarly, nodal upstaging was an increase in staging from any initial clinical N stage to surgical pathologic N stage as N0, N1, N2, N3. T and N staging were considered separately as upstaging of the primary tumor and lymph nodes often do not occur in tandem. Separate consideration of T and N was felt to provide greater specificity about tumor changes and the impact of longer time intervals on tumor growth and behavior.

We examined upstaging as a binary variable (any vs none), and by degree of upstaging (i.e. one stage increase vs 2+ stages increased). We assumed that upstaging can be due to clinical understaging due to the inaccuracies of imaging and/or physical examination, as well as tumor growth and nodal disease spread. Clinical stage is not completely accurate, and we tried to minimize potential bias by excluding patients without biopsy-based staging or histologic diagnostic confirmation. Because downstaging from clinical inaccuracy is less of a concern, upstaging was modeled as a binary function as present or not, with downstaging included as part of the group that was not upstaged.

**Upstaging Due to Delay-Related Growth:** To examine upstaging due to inaccurate clinical staging separately from delay-related growth or disease advancement, we assessed upstaging rates in patients having surgery ≤15 days from diagnosis, assuming negligible growth within that time. Consequently, the rate of upstaging due to delay is the rate of upstaging overall, less that rate of inaccuracy at diagnosis.

**Potential Confounders**

Patient demographic and tumor characteristics were selected as potential confounders by the primary investigator based on literature and experience. These included clinical T and N stage, phenotype, histology, age at diagnosis, sex, race, Hispanic ethnicity, and grade.

**Clinical T and N Stages:** For T upstaging, we ran separate models for each clinical T stage (Any cTis, cT1, and cT2), so covariate adjustment was based on the observed covariate values for patients within cT stage. Covariates are delineated below. In a separate model for nodal upstaging, we included patients who were cN0, so that the outcome could be interpreted as upstaging to node positivity (pN1, pN2 or pN3), which is more clinically relevant than upstaging from N1 or N2. This model included cT stage instead of cN stage as a covariate. The logistic model results were presented as adjusted predicted probabilities of upstaging for delay (continuous) at 30-day intervals, as well as the corresponding odds ratio for a difference of 30 days.

The likelihood of upstaging is related to the initial clinical T or N stage, and in our analyses we stratified for clinical T and N staging, or included these as categorical variables for covariate adjustment where cN would be included as a covariate for T-upstaging models, and cT would be included as a covariate for nodal upstaging.

**Phenotype:** Since phenotype is an important determinant of both tumor behavior and first-course treatment of invasive cancer, we evaluated the association of delay and T stage upstaging in subset analyses that excluded ductal carcinoma *in situ* (DCIS) and those of unknown phenotypes. Odds ratio estimates for phenotypes were from three separate logistic regression models, one for each phenotype, each adjusting for the same covariates (see below).

Hormone receptor positive (HR+) tumors were defined as those that were estrogen receptor (ER) and/or progesterone receptor (PR) positive, using the threshold of positivity defined at the time of diagnosis, and human epidermal growth factor receptor 2 (HER2)-negative. Immunohistochemically-negative HER2 staining was defined as a score of 0 or 1+. Triple negative (TN) breast cancers were defined as those being ER–, PR– and HER2–, and HER2+ patients could have any ER and/or PR status. Patients with missing or unknown receptor status were included as a separate category. Phenotype (as HR+, HER2+, TN, and unknown) was included for invasive cancer models, while hormone receptor status was a covariate for DCIS, when HER2 status is usually not assessed.

**Histology:** Histology was categorized as ductal, lobular, or other, based on the ICD-O codes included in the NCDB database. To explore whether invasive lobular carcinomas are more likely to be upstaged, as they are more difficult than invasive ductal carcinomas to see on imaging and palpate on examination, in sensitivity analyses we stratified by invasive ductal vs invasive lobular histology.

**Grade:** Grade was based on the pathologic grade, which was available for all years in the analysis. Grade was included as a categorical variable with 4 levels, including (1) Well Differentiated/Grade 1/ Low Nuclear Grade; (2) Moderately Differentiated/ Grade 2/ Intermediate Nuclear Grade; (3) Poorly Differentiated/Grade 3/High Nuclear Grade or Undifferentiated, and (4) Grade Unknown/Not Available/Missing, where poorly differentiated and undifferentiated were combined due to small numbers.

**Other Potential Confounders**: In all multivariable logistic models, we included age at diagnosis (years) as a continuous variable, sex (categorical), race, and Hispanic ethnicity. In the NCDB, race is pulled from the medical record and is reported as recorded in cancer registries, by patient self-reporting. For this study, there were four race categories: ”White” and “Black,” which each include only one race code; “Asian” includes 25 NCDB race codes; and “Other/Unknown” includes the codes: “American Indian, Aleutian, or Eskimo,” “Other,” and “Unknown.” Hispanic ethnicity was classified as “yes” to include the NCDB’s categories of: Mexican (including Chicana); Puerto Rican; Cuban; South or Central American (excluding Brazilian); Spanish, Hispanic or Latino NOS; Dominican; Spanish surname; and other unclassified Hispanic origin, while “no” included only “Non-Spanish, Non-Hispanic,” and “unknown” included “Unknown whether of Spanish/Hispanic origin; not stated in patient record.”

**Statistical Analyses**

We described patient demographic, tumor, and treatment characteristics of the analytic cohort with frequencies and percents, or medians and interquartile ranges (IQRs), by clinical T stage (Any cTis, cT1, and cT2) and by clinical N stage (cN0, cN1, and cN2) categories. We summarized the delay interval (days from diagnosis to first or only surgery) using descriptive statistics (means, standard deviations, medians, interquartile ranges [IQRs]) by T-stage upstaging within clinical T stage and phenotype subgroups, and with histograms. For any cTis and cT1 stages, we examined delay intervals for increasing by one stage only (e.g. Any cTis to pT1) and more than one stage (e.g. Any cTis to pT2 or pT3).

**Primary Hypothesis of Delay and Upstaging:** Our primary hypothesis was that longer surgical delays are associated with growth, demonstrable by an increase in the likelihoods of T and N stage upstaging. We examined the diagnosis to surgery interval as a categorical and a continuous variable. Categorical intervals were set at 30 days to remain consistent with our prior work exploring survival declines due to delays (Bleicher, JAMA Oncol 2016), and because this is an easily-referenced category. We initially examined the proportion of upstaged patients in 30-day delay intervals beyond the first 15-day period (1-15, 16-45, 46-75, etc.) by clinical T stage group for T upstaging, and by clinical N stage group for nodal upstaging (data not shown). An increasing pattern of upstaging was observed in clinical T groups that was consistent with a logit model.

For tumor upstaging and nodal upstaging separately, we examined the associations between delay and upstaging (yes vs no) using multivariable logistic regression models with robust standard errors clustered by facility. In these models, the delay interval was included as a continuous variable (days), and the odds ratio was estimated for a 30-day difference in delays. Covariate adjustment was based on the observed values for patients within each subgroup. We included potential confounders including age, sex, race, Hispanic ethnicity, grade and histology in all multivariable models; we included additional key explanatory variables depending on the focus of the subgroup analyses. We obtained average adjusted predicted probabilities of upstaging at specified delay intervals of 15 days (baseline inaccuracies), 30 days, 60 days, 90 days, 120 days, 150 days, and 180 days. These predicted probabilities were used to compute the delay-attributable risk of upstaging by subtracting the baseline inaccuracies estimate from the estimates at the 30-day intervals.

We estimated covariate adjusted probabilities and delay-attributable probabilities for the T-upstaging for the subgroups: cTis (N0 only), cT1, cT2, and by phenotype (HR+, HER2+, TN) with cTis excluded. We also modeled nodal upstaging for cN0 patients at the same delay timepoints. In sensitivity analyses, we estimated upstaging probabilities for lobular and ductal separately within the previous subgroups.

**Secondary Hypothesis of Delay and Linear Dimension Tumor Growth Rate:** The NCDB has variables for pathologic stage and pathologic tumor size, as well as clinical stage, but does not have a variable for clinical tumor size. Ideal regressions to determine growth rates would contain highly-accurate starting sizes and final sizes surrounding the preoperative delay with sufficient observations to make a meaningful estimate. Alternatively, clinical T stage provides a size range which can be used for starting size. Since clinical estimates of tumor size are known to be imprecise and heterogeneous in their accuracy and precision (depending on how that size is estimated), these caveats were felt to make the existing groupings within clinical T stage reasonable and consistent with real-world practice.

We inferred growth rates of tumors from analyses using the regression of pathologic tumor size on delay interval as a continuous variable. For these analyses, we excluded patients with DCIS (where size is not usually measured), and any tumor whose size was missing or zero, or any patients with missing phenotype. Consistent with the determination that tumors having times between diagnosis and surgery ≤15 days are considered to have negligible growth, tumors whose delay was ≤15 days were also excluded to more accurately assess growth rates. For these analyses, we used separate subcategories for clinical T1 stage (cT = cT1a, cT1b, cT1c, cT1 NOS, and cT2) as these reflect the initial tumor size and allow the narrowest clinical stage groupings (i.e. cT1a and cT1b are 5 mm ranges, cT1c is a 10 mm range) for homogeneity within each regression’s individual cohort. cT1mi was excluded because of the lack of precise size needed for regression.

For estimates of linear growth rates over time, within each clinical T stage category (cT1a, cT1b, cT1c, cT1NOS, and cT2), we assumed that growth rates are constant, consistent with the concept that larger tumors are growing faster linearly. In the general linear regression model, we included an interaction term between delay (continuous) and clinical T subcategory to determine if the slope of tumor size on delay differed by clinical T category; to obtain unconditional slope estimates, we did not include other covariates. We utilized the slope of tumor size on delay (in mm/30 day intervals) to be the estimate of the change in tumor size over time, within each clinical T subcategory. We interpreted these results as growth rates by taking the reciprocal of these slopes, i.e. slope as (mm/30 days) 🡪 growth rates as (30 days/mm), and used the reciprocals of the regression coefficient 95% confidence intervals as the growth rate confidence interval.

Assuming the growth rates within each cT-stage extend beyond 180-day delays, we created a figure with concatenated curves to illustrate progressively increasing rates associated with tumor enlargement. We used the growth rate slopes estimated from regression analysis to extrapolate each growth curve on a hypothetical timeline to reach the next stage. This offers a visual perspective showing consistency with prior theories on increasing tumor growth rates with size (see manuscript reference 18, Vonfournier et al, *Cancer* 1980).

**Statistical Significance and Software:** In this large cohort, statistical significance should not be the basis for interpreting the results. All tests were two-sided with 1% type I error instead of 5%, given the large sample size, but clinical significance was still the primary consideration in light of the large sample size for all outcomes. Analyses were conducted using SAS 9.4 (SAS Institute, Cary NC) and Stata 15 (StataCorp, College Station, TX), and figures were created using SAS, Stata, and Excel.
