# Supplementary material for: Breast Cancer Upstaging Risk and In Vivo Tumor Growth Rates Associated with Preoperative Delays
Source: Ann Surg Oncol. 2025 Jul 23;32(12):8789–97. doi: 10.1245/s10434-025-17867-9 (PMC12534249; doi:10.1245/s10434-025-17867-9)
Supplement: Supplementary file 2 — Supplementary file2 (DOCX 101 KB) [file 10434_2025_17867_MOESM2_ESM.docx]

**Supplementary Table 1: Exclusions to create the analytic dataset with specifics by variable. Starting cohort included patients treated 2010-2020 (n=2,594,500)**

| Exclusion specifics | STROBE items | Frequency | Percent | Cumulative Frequency | Cumulative Percent |
| --- | --- | --- | --- | --- | --- |
| 01: Not 1st cancer (SEQUENCE_NUMBER ne 00,01) |  | 493945 | 19.04 | 493945 | 19.04 |
| 02: Diagnostic Confirmation was not from positive histology (DIAGNOSTIC_CONFIRMATION) |  | 12038 | 0.46 | 505983 | 19.50 |
| 03: Diagnostic procedure was not a biopsy (RX_SUMM_DXSTG_PROC) |  | 199255 | 7.68 | 705238 | 27.18 |
| 04: No surgery performed (primary site surgery = 00) |  | 139730 | 5.39 | 844968 | 32.57 |
| 05: Surgery type was local destruction, NOS or unknown |  | 7110 | 0.27 | 852078 | 32.84 |
| 06: Diagnosis ONLY at the reporting facility (CLASS_OF_CASE = 00) |  | 43418 | 1.67 | 895496 | 34.52 |
| 07: Palliative care given or unknown (PALLIATIVE_CARE) |  | 3764 | 0.15 | 899260 | 34.66 |
|  |  |  |  |  |  |
| Z:INCLUDED | **Box 1, initial** | **1695240** | **65.34** | **2594500** | **100.00** |
|  |  |  |  |  |  |
| Exclusion specifics | N excluded | Frequency | Percent | Cumulative Frequency | Cumulative Percent |
| 08a: Clinical Stage 4 (TNM_CLIN_STAGE_GROUP) | 17444 | 10294 | 0.61 | 10294 | 0.61 |
| 08b: Pathologic Stage 4 (TNM_PATH_STAGE_GROUP=4) |  | 1741 | 0.10 | 12035 | 0.71 |
| 08c: Metastasis (TNM_PATH_M=p1/TNM_CLIN_M=c1,c01+) |  | 540 | 0.03 | 12575 | 0.74 |
| 08d: Metastases of bone 2016-18 (METS_AT_DX_BON 1,8,9) |  | 2658 | 0.16 | 15233 | 0.90 |
| 08d: Metastases of brain 2016-18 (METS_AT_DX_BRN 1,8,9) |  | 103 | 0.01 | 15336 | 0.90 |
| 08d: Metastases of distant lymph nodes 2016-18 (METS_AT_DX_DLN 1,8,9) |  | 774 | 0.05 | 16110 | 0.95 |
| 08d: Metastases of liver 2016-18 (METS_AT_DX_LIV 1,8,9) |  | 381 | 0.02 | 16491 | 0.97 |
| 08d: Metastases of lung 2016-18 (METS_AT_DX_LNG 1,8,9) |  | 385 | 0.02 | 16876 | 1.00 |
| 08d: Metastases of other site 2016-18 (METS_AT_DX_OTH 1,8,9) |  | 233 | 0.01 | 17109 | 1.01 |
| 08e: Analytic stage group = IV |  | 335 | 0.02 | 17444 | 1.03 |
| 09a: Inflammatory breast cancer (AJCC_TNM_CLIN_T T4d) | 10415 | 2357 | 0.14 | 19801 | 1.17 |
| 09b: Inflammatory breast cancer (AJCC_TNM_PATH_T T4d) |  | 49 | 0.00 | 19850 | 1.17 |
| 09c: Inflammatory breast cancer (TNM_CLIN_T T4d) |  | 6462 | 0.38 | 26312 | 1.55 |
| 09d: Inflammatory breast cancer (TNM_path_T T4d) |  | 613 | 0.04 | 26925 | 1.59 |
| 09e: Inflammatory breast cancer (CS extension = 600,710,715,720,725,730,750,780) |  | 934 | 0.06 | 27859 | 1.64 |
| 10a: Lobular carcinoma *in situ* (hist_gp and BEHAVIOR) | 20815 | 18952 | 1.12 | 46811 | 2.76 |
| 10b: Ductal Carcinoma and Lobular Carcinoma *In Situ* |  | 1863 | 0.11 | 48674 | 2.87 |
| 11: Tumor size = diffuse (code=998) | 711 | 711 | 0.04 | 49385 | 2.91 |
| 12a: cT4, cT4a, cT4b, cT4c | 21938 | 18187 | 1.07 | 67572 | 3.99 |
| 12b: pT4, pT4a, pT4b, pT4c |  | 3751 | 0.22 | 71323 | 4.21 |
| 13a: cT0 | 2112 | 2112 | 0.12 | 73435 | 4.33 |
| 13b: pT0 | 34506 | 34506 | 2.04 | 107941 | 6.37 |
| 14a: cT or pT missing | 296731 | 192611 | 11.36 | 300552 | 17.73 |
| 14b: cN or pN missing |  | 104061 | 6.14 | 404613 | 23.87 |
| 14c: TNM edition not edition 7 or 8 (TNM_EDITION_NUMBER) |  | 59 | 0.00 | 404672 | 23.87 |
| 15: Analytic stage grouping unknown | 2735 | 2735 | 0.16 | 407407 | 24.03 |
| 16a: cN3 any (thus no upstage possible) | 5674 | 5674 | 0.33 | 413081 | 24.37 |
| 16b: cT3 any (thus no upstage possible) | 42181 | 42181 | 2.49 | 455262 | 26.86 |
|  |  |  |  |  |  |
| Z:INCLUDED |  | **1239978** | **73.14** | **1695240** | **100.00** |
|  |  |  |  |  |  |
| Exclusion specifics | N excluded | Frequency | Percent | Cumulative Frequency | Cumulative Percent |
| 17a: Diagnosis to surgery = 0 days (DX_SURG1_DAYS=0) | 36728 | 36728 | 2.96 | 36728 | 2.96 |
| 17b: Days from diagnosis-to-surgery AND definitive surgery missing or 0 (dx_surg1_days,DX_SURG_STARTED_DAYS) | 6183 | 6183 | 0.50 | 42911 | 3.46 |
| 17c: Diagnosis to surgery > 180 days | 47463 | 31949 | 2.58 | 74860 | 6.04 |
| 17d: Diagnosis to 2^nd^ or definitive surgery > 180 days |  | 15514 | 1.25 | 90374 | 7.29 |
| 17e: Days from diagnosis to diagnostic procedure > days from diagnosis to definitive surgery | 1023 | 1023* | 0.08* | 91397* | 7.37* |
| 17f: Days from diagnosis-to-surgery > Days from diagnosis-to-definitive-surgery |  | * | * | * | * |
| Z:INCLUDED |  | **1148581** | **92.63** | **1239978** | **100.00** |
|  |  |  |  |  |  |
| Exclusion specifics | N excluded | Frequency | Percent | Cumulative Frequency | Cumulative Percent |
| 18: Neoadjuvant chemotherapy (DX_CHEMO_STARTED_DAYS<DX_SURG1_DAYS) | 127735 | 21149 | 1.84 | 21149 | 1.84 |
| 19: Neoadjuvant radiotherapy (DX_RAD_STARTED_DAYS<DX_SURG1_DAYS var) |  | 583 | 0.05 | 21732 | 1.89 |
| 20a: Neoadjuvant hormonal therapy (DX_HORMONE_STARTED_DAYS) |  | 13448 | 1.17 | 35180 | 3.06 |
| 20b: Neoadjuvant immunotherapy (DX_IMMUNO_STARTED_DAYS) |  | 138 | 0.01 | 35318 | 3.07 |
| 20c: Neoadjuvant other therapy (DX_OTHER_STARTED_DAYS) |  | 169 | 0.01 | 35487 | 3.09 |
| 20d: Neoadjuvant systemic treatment (DX_SYSTEMIC_STARTED_DAYS) |  | 35 | 0.00 | 35522 | 3.09 |
| 20e: Neoadjuvant any treatment (DX_RX_STARTED_DAYS) |  | 46 | 0.00 | 35568 | 3.10 |
| 20f: Unknown chemotherapy, (RX_SUMM_CHEMO 88,99) |  | 8360 | 0.73 | 43928 | 3.82 |
| 20g: Chemotherapy given, days missing (Rx_SUMM_CHEMO 1,2,3) |  | 5767 | 0.50 | 49695 | 4.33 |
| 20h: Unknown immunotherapy (Rx_SUMM_IMMUNO 88,99) |  | 1189 | 0.10 | 50884 | 4.43 |
| 20i: Immunotherapy given, days missing (RX_SUMM_HORM 1) |  | 225 | 0.02 | 51109 | 4.45 |
| 21a: Neoadjuvant per radiotherapy-surgery sequence or unknown |  | 27056 | 2.36 | 78165 | 6.81 |
| 21b: Neoadjuvant or intraop chemotherapy (RX_SUMM_SYSTEMIC_SUR_SEQ) or unknown |  | 45746 | 3.98 | 123911 | 10.79 |
| 21c: Other treatment not NONE |  | 2319 | 0.20 | 126230 | 10.99 |
| 21d: Hematologic transplant & endocrine procedures |  | 1483 | 0.13 | 127713 | 11.12 |
| 21e: Treatment not recorded as GIVEN |  | 22 | 0.00 | 127735 | 11.12 |
| 22a: No mass/tumor found (TUMOR_SIZE=0) | 2479 | 40 | 0.00 | 127775 | 11.12 |
| 22b: No mass/tumor found 2016+ |  | 139 | 0.01 | 127914 | 11.14 |
| 22c: Tumor size missing** |  | ** | ** | ** | ** |
| 22d: Tumor size unknown, not DCIS** |  | 2230 | 0.20 | 130214 | 11.34 |
| 23: Tumor size > 500 mm | 148 | 148 | 0.01 | 130362 | 11.35 |
|  |  |  |  |  |  |
| Z:INCLUDED |  | **1018219** | **88.65** | **1148581** | **100.00** |

NOS = not otherwise specified

*17f: Days from diagnosis-to-surgery > Days from diagnosis-to-definitive-surgery was combined with line item 17e: Days from diagnosis to diagnostic procedure > days from diagnosis to definitive surgery so that a group <10 was censored and thus incalculable.

**22c: Tumor size missing was combined with 22d: Tumor size unknown, not DCIS so that a group <10 was censored and thus incalculable.

**Supplementary Table 2. Cohort characteristics by T stage. Numbers represent *n* (%).**

|  | All | cTis | cT1 | cT2 |
| --- | --- | --- | --- | --- |
| All | 1,018,219 (100.0) | 187,850 (100.0) | 641,651 (100.0) | 188,718 (100.0) |
| Age at Diagnosis |  |  |  |  |
| 18-39 | 31,660 (3.1) | 6,269 (3.3) | 15,309 (2.4) | 10,082 (5.3) |
| 40-49 | 156,386 (15.4) | 37,276 (19.8) | 86,731 (13.5) | 32,379 (17.2) |
| 50-59 | 253,673 (24.9) | 51,730 (27.5) | 156,570 (24.4) | 45,373 (24.0) |
| 60-69 | 312,719 (30.7) | 55,174 (29.4) | 207,580 (32.4) | 49,965 (26.5) |
| 70-79 | 200,938 (19.7) | 30,522 (16.2) | 136,720 (21.3) | 33,696 (17.9) |
| 80+ | 62,843 (6.2) | 6,879 (3.7) | 38,741 (6.0) | 17,223 (9.1) |
| Sex |  |  |  |  |
| Female | 1,011,105 (99.3) | 187,175 (99.6) | 637,816 (99.4) | 186,114 (98.6) |
| Male | 7,114 (0.7) | 675 (0.4) | 3,835 (0.6) | 2,604 (1.4) |
| Race |  |  |  |  |
| White | 847,611 (83.2) | 147,214 (78.4) | 546,141 (85.1) | 154,256 (81.7) |
| Black | 105,868 (10.4) | 25,395 (13.5) | 58,787 (9.2) | 21,686 (11.5) |
| Asian | 42,922 (4.2) | 10,790 (5.7) | 23,574 (3.7) | 8,558 (4.5) |
| Other/Unknown | 21,818 (2.1) | 4,451 (2.4) | 13,149 (2.0) | 4,218 (2.2) |
| Hispanic Ethnicity |  |  |  |  |
| No | 938,314 (92.2) | 171,892 (91.5) | 594,275 (92.6) | 172,147 (91.2) |
| Yes | 56,466 (5.5) | 12,019 (6.4) | 32,409 (5.1) | 12,038 (6.4) |
| Unknown | 23,439 (2.3) | 3,939 (2.1) | 14,967 (2.3) | 4,533 (2.4) |
| Charlson Score |  |  |  |  |
| 0 | 843,044 (82.8) | 157,996 (84.1) | 531,897 (82.9) | 153,151 (81.2) |
| 1 | 132,961 (13.1) | 22,894 (12.2) | 83,721 (13.0) | 26,346 (14.0) |
| ≥2 | 42,214 (4.1) | 6,960 (3.7) | 26,033 (4.1) | 9,221 (4.9) |
| Insurance |  |  |  |  |
| Private | 539,900 (53.0) | 111,489 (59.4) | 331,792 (51.7) | 96,619 (51.2) |
| Medicaid | 55,726 (5.5) | 10,126 (5.4) | 31,537 (4.9) | 14,063 (7.5) |
| Medicare | 388,490 (38.2) | 59,614 (31.7) | 258,310 (40.3) | 70,566 (37.4) |
| Other Governmental, Uninsured, Unknown | 34,103 (3.3) | 6,621 (3.5) | 20,012 (3.1) | 7,470 (4.0) |
| Had A Second Cancer Subsequently |  |  |  |  |
| No | 927,021 (91.0) | 171,454 (91.3) | 585,901 (91.3) | 169,666 (89.9) |
| Yes | 91,198 (9.0) | 16,396 (8.7) | 55,750 (8.7) | 19,052 (10.1) |
| Surgery Not Performed at Reporting Hospital |  |  |  |  |
| No | 874,857 (85.9) | 167,620 (89.2) | 545,457 (85.0) | 161,780 (85.7) |
| Yes | 143,362 (14.1) | 20,230 (10.8) | 96,194 (15.0) | 26,938 (14.3) |
| Median Income Quartile |  |  |  |  |
| Quartile 1 (lowest median income) | 120,498 (11.8) | 21,987 (11.7) | 73,330 (11.4) | 25,181 (13.3) |
| Quartile 2 | 174,611 (17.1) | 30,543 (16.3) | 109,873 (17.1) | 34,195 (18.1) |
| Quartile 3 | 216,631 (21.3) | 38,345 (20.4) | 137,217 (21.4) | 41,069 (21.8) |
| Quartile 4 (highest median income) | 366,859 (36.0) | 70,142 (37.3) | 233,540 (36.4) | 63,177 (33.5) |
| Missing/unknown | 139,620 (13.7) | 26,833 (14.3) | 87,691 (13.7) | 25,096 (13.3) |
| No High School Diploma |  |  |  |  |
| Quartile 1 (most with no diploma) | 138,079 (13.6) | 26,399 (14.1) | 82,940 (12.9) | 28,740 (15.2) |
| Quartile 2 | 211,328 (20.8) | 38,064 (20.3) | 131,944 (20.6) | 41,320 (21.9) |
| Quartile 3 | 270,983 (26.6) | 48,580 (25.9) | 172,415 (26.9) | 49,988 (26.5) |
| Quartile 4 (least with no diploma) | 259,298 (25.5) | 48,139 (25.6) | 167,373 (26.1) | 43,786 (23.2) |
| Missing/unknown | 138,531 (13.6) | 26,668 (14.2) | 86,979 (13.6) | 24,884 (13.2) |
| Cancer Program Type* |  |  |  |  |
| Community | 69,774 (7.1) | 10,919 (6.0) | 45,219 (7.2) | 13,636 (7.6) |
| Comprehensive Community | 413,606 (41.9) | 73,620 (40.5) | 263,986 (42.1) | 76,000 (42.5) |
| Academic/Research | 287,329 (29.1) | 55,205 (30.4) | 181,042 (28.9) | 51,082 (28.6) |
| Integrated Network | 215,850 (21.9) | 41,837 (23.0) | 136,095 (21.7) | 37,918 (21.2) |
| Histology |  |  |  |  |
| Ductal | 921,060 (90.5) | 187,369 (99.7) | 573,586 (89.4) | 160,105 (84.8) |
| Lobular | 88,381 (8.7) | 0 0 | 63,383 (9.9) | 24,998 (13.2) |
| Other | 8,778 (0.9) | 481 (0.3) | 4,682 (0.7) | 3,615 (1.9) |
| Grade^#^ |  |  |  |  |
| Well Diff/ Grade 1/ Low Nuclear Gr | 256,906 (25.2) | 20,813 (11.1) | 207,384 (32.3) | 28,709 (15.2) |
| Moderately Diff/ Grade 2/ Intermediate Nuclear Gr | 451,446 (44.3) | 68,041 (36.2) | 295,971 (46.1) | 87,434 (46.3) |
| Poorly Diff/Grade 3/High Nuclear Gr/Undifferentiated | 251,547 (24.7) | 75,706 (40.3) | 110,880 (17.3) | 64,961 (34.4) |
| Grade Unknown/Not Available/Missing | 256,906 (25.2) | 20,813 (11.1) | 207,384 (32.3) | 28,709 (15.2) |
| Clinical Stage |  |  |  |  |
| 0 | 187,443 (18.4) | 186,748 (99.4) | 626 (0.1) | 69 (0.0) |
| 1 | 24,121 (2.4) | 62 (0.0) | 23,693 (3.7) | 366 (0.2) |
| 1A | 579,069 (56.9) | 183 (0.1) | 576,474 (89.8) | 2,412 (1.3) |
| 1B | 43,032 (4.2) | *** | *** | 25,985 (13.8) |
| 2 | 3,880 (0.4) | *** | *** | 3,479 (1.8) |
| 2A | 142,591 (14.0) | 39 (0.0) | 16,109 (2.5) | 126,443 (67.0) |
| 2B | 24,916 (2.4) | *** | *** | 24,323 (12.9) |
| 3 | 163 (0.0) | 0 (0) | 30 (0.0) | 133 (0.1) |
| 3A | 4,042 (0.4) | *** | *** | 2,956 (1.6) |
| 3B | 362 (0.0) | 0 (0) | 17 (0.0) | 345 (0.2) |
| 3C | 82 (0.0) | 0 (0) | 16 (0.0) | 66 (0.0) |
| Unknown/Missing | 8,518 (0.8) | 804 (0.4) | 5,573 (0.9) | 2,141 (1.1) |
| ER Status |  |  |  |  |
| Negative | 126,373 (12.4) | 31,296 (16.7) | 65,106 (10.1) | 29,971 (15.9) |
| Positive | 884,220 (86.8) | 151,458 (80.6) | 574,600 (89.6) | 158,162 (83.8) |
| Missing/Unknown/Borderline | 7,626 (0.7) | 5,096 (2.7) | 1,945 (0.3) | 585 (0.3) |
| PR Status |  |  |  |  |
| Negative | 209,906 (20.6) | 46,087 (24.5) | 116,098 (18.1) | 47,721 (25.3) |
| Positive | 785,477 (77.1) | 122,582 (65.3) | 522,673 (81.5) | 140,222 (74.3) |
| Missing/Unknown/Borderline | 22,836 (2.2) | 19,181 (10.2) | 2,880 (0.4) | 775 (0.4) |
| HER2 Status |  |  |  |  |
| Negative | 777,403 (76.3) | 27,053 (14.4) | 582,510 (90.8) | 167,840 (88.9) |
| Positive | 63,713 (6.3) | 10,708 (5.7) | 37,996 (5.9) | 15,009 (8.0) |
| Missing/Unknown/Borderline | 177,103 (17.4) | 150,089 (79.9) | 21,145 (3.3) | 5,869 (3.1) |
| Phenotype |  |  |  |  |
| ER+/PR+, HER2-** | 705,243 (69.3) | 23,590 (12.6) | 535,803 (83.5) | 145,850 (77.3) |
| HER2+** | 63,427 (6.2) | 10,583 (5.6) | 37,878 (5.9) | 14,966 (7.9) |
| TN** | 70,832 (7.0) | 3,231 (1.7) | 45,876 (7.1) | 21,725 (11.5) |
| Missing//Unknown/Borderline | 28,271 (2.8) | 0 (0) | 22,094 (3.4) | 6,177 (3.3) |
| Tis ER+ or PR+ | 123,719 (12.2) | 123,719 (65.9) | 0 (0) | 0 (0) |
| Tis ER-/PR- | 19,844 (1.9) | 19,844 (10.6) | 0 (0) | 0 (0) |
| Tis ER and PR Missing | 4,983 (0.5) | 4,983 (2.7) | 0 (0) | 0 (0) |
| Tis ER- or PR-, Other Missing | 1,900 (0.2) | 1,900 (1.0) | 0 (0) | 0 (0) |
| cT Stage |  |  |  |  |
| Any Tis | 187,850 (18.4) | 187,850 (100) | 0 (0) | 0 (0) |
| cT1mi | 7,652 (0.8) | 0 (0) | 7,652 (1.2) | 0 (0) |
| cT1a | 58,399 (5.7) | 0 (0) | 58,399 (9.1) | 0 (0) |
| cT1b | 187,893 (18.5) | 0 (0) | 187,893 (29.3) | 0 (0) |
| cT1c | 280,982 (27.6) | 0 (0) | 280,982 (43.8) | 0 (0) |
| cT1, Not Otherwise Specified | 106,725 (10.5) | 0 (0) | 106,725 (16.6) | 0 (0) |
| cT2 | 188,718 (18.5) | 0 (0) | 0 (0) | 188,718 (100.0) |
| cN Stage |  |  |  |  |
| cN0 | 970,778 (95.3) | 187,734 (99.9) | 621,436 (96.8) | 161,608 (85.6) |
| cN1 | 43,206 (4.2) | 105 (0.1) | 18,971 (3.0) | 24,130 (12.8) |
| cN2 | 4,235 (0.4) | 11 (0.0) | 1,244 (0.2) | 2,980 (1.6) |
| Surgery Type |  |  |  |  |
| Breast Conservation | 686,855 (67.5) | 116,204 (61.9) | 478,666 (74.6) | 91,985 (48.7) |
| Mastectomy | 331,364 (32.5) | 71,646 (38.1) | 162,985 (25.4) | 96,733 (51.3) |
| Nodal Procedure^∞^ |  |  |  |  |
| None | 87,081 (8.6) | 76,310 (40.6) | 8,604 (1.3) | 2,167 (1.1) |
| Performed, But Not Otherwise Specified | 147,752 (14.5) | 17,085 (9.1) | 96,994 (15.1) | 33,673 (17.8) |
| Sentinel Node Biopsy (or Excisional Biopsy) | 602,132 (59.1) | 77,680 (41.4) | 428,011 (66.7) | 96,441 (51.1) |
| ALND | 67,446 (6.6) | 6,913 (3.7) | 37,895 (5.9) | 22,638 (12.0) |
| Sentinel Node Biopsy and ALND Simultaneously | 103,923 (10.2) | 9,107 (4.8) | 64,381 (10.0) | 30,435 (16.1) |
| Sentinel Node Biopsy and Subsequent ALND | 9,597 (0.9) | 612 (0.3) | 5,654 (0.9) | 3,331 (1.8) |
| Unknown | 288 (0.0) | 143 (0.1) | 112 (0.0) | 33 (0.0) |

*Cancer program type variable in the NCDB excludes patients<40. This variable includes a subset of 986,559 patients.

^#^Variables in different years that combines differentiation, grade, and nuclear grade are combined. Diff = differentiated, Gr = grade. Grade defined as clinical grade where available (2018+) and pathologic grade otherwise.

**ER = estrogen receptor-positive, PR = progesterone receptor-positive, HER2 = human epidermal growth factor 2-positive. Phenotype for invasive disease for these categories requires complete data for ER, PR and HER2.
***Cells <10 or those enabling calculations of those cells have been censored as per the requirements of the National Cancer Database.

^∞^ALND = Axillary lymph node dissection (any number of nodes)

**Supplementary Table 3. Cohort characteristics by N stage. Numbers represent *n* (%).**

|  | All | cN0 | cN1 | cN2 |
| --- | --- | --- | --- | --- |
| All | 1,018,219 (100.0) | 970,778 (100.0) | 43,206 (100.0) | 4,235 (100.0) |
| Age at Diagnosis |  |  |  |  |
| 18-39 | 31,660 (3.1) | 28,700 (3.0) | 2,689 (6.2) | 271 (6.4) |
| 40-49 | 156,386 (15.4) | 147,791 (15.2) | 7,872 (18.2) | 723 (17.1) |
| 50-59 | 253,673 (24.9) | 241,427 (24.9) | 11,156 (25.8) | 1,090 (25.7) |
| 60-69 | 312,719 (30.7) | 300,654 (31.0) | 11,022 (25.5) | 1,043 (24.6) |
| 70-79 | 200,938 (19.7) | 193,307 (19.9) | 6,932 (16.0) | 699 (16.5) |
| 80+ | 62,843 (6.2) | 58,899 (6.1) | 3,535 (8.2) | 409 (9.7) |
| Sex |  |  |  |  |
| Female | 1,011,105 (99.3) | 964,385 (99.3) | 42,559 (98.5) | 4,161 (98.3) |
| Male | 7,114 (0.7) | 6,393 (0.7) | 647 (1.5) | 74 (1.7) |
| Race |  |  |  |  |
| White | 847,611 (83.2) | 810,232 (83.5) | 34,021 (78.7) | 3,358 (79.3) |
| Black | 105,868 (10.4) | 98,858 (10.2) | 6,393 (14.8) | 617 (14.6) |
| Asian | 42,922 (4.2) | 40,965 (4.2) | 1,791 (4.1) | 166 (3.9) |
| Other/Unknown | 21,818 (2.1) | 20,723 (2.1) | 1,001 (2.3) | 94 (2.2) |
| Hispanic Ethnicity |  |  |  |  |
| No | 938,314 (92.2) | 895,748 (92.3) | 38,827 (89.9) | 3,739 (88.3) |
| Yes | 56,466 (5.5) | 53,058 (5.5) | 3,085 (7.1) | 323 (7.6) |
| Unknown | 23,439 (2.3) | 21,972 (2.3) | 1,294 (3.0) | 173 (4.1) |
| Charlson Score |  |  |  |  |
| 0 | 843,044 (82.8) | 804,512 (82.9) | 35,079 (81.2) | 3,453 (81.5) |
| 1 | 132,961 (13.1) | 126,271 (13.0) | 6,083 (14.1) | 607 (14.3) |
| ≥2 | 42,214 (4.1) | 39,995 (4.1) | 2,044 (4.7) | 175 (4.1) |
| Insurance |  |  |  |  |
| Private | 539,900 (53.0) | 515,188 (53.1) | 22,609 (52.3) | 2,103 (49.7) |
| Medicaid | 55,726 (5.5) | 51,564 (5.3) | 3,760 (8.7) | 402 (9.5) |
| Medicare | 388,490 (38.2) | 372,053 (38.3) | 14,920 (34.5) | 1,517 (35.8) |
| Other Governmental, Uninsured, Unknown | 34,103 (3.3) | 31,973 (3.3) | 1,917 (4.4) | 213 (5.0) |
| Had A Second Cancer Subsequently |  |  |  |  |
| No | 927,021 (91.0) | 884,565 (91.1) | 38,670 (89.5) | 3,786 (89.4) |
| Yes | 91,198 (9.0) | 86,213 (8.9) | 4,536 (10.5) | 449 (10.6) |
| Surgery Not Performed at Reporting Hospital | |  |  |  |
| No | 874,857 (85.9) | 835,063 (86.0) | 36,373 (84.2) | 3,421 (80.8) |
| Yes | 143,362 (14.1) | 135,715 (14.0) | 6,833 (15.8) | 814 (19.2) |
| Median Income Quartile |  |  |  |  |
| Quartile 1 (lowest median income) | 120,498 (11.8) | 113,546 (11.7) | 6,261 (14.5) | 691 (16.3) |
| Quartile 2 | 174,611 (17.1) | 165,798 (17.1) | 7,938 (18.4) | 875 (20.7) |
| Quartile 3 | 216,631 (21.3) | 206,144 (21.2) | 9,536 (22.1) | 951 (22.5) |
| Quartile 4 (highest median income) | 366,859 (36.0) | 351,437 (36.2) | 14,137 (32.7) | 1,285 (30.3) |
| Missing/unknown | 139,620 (13.7) | 133,853 (13.8) | 5,334 (12.3) | 433 (10.2) |
| No High School Diploma |  |  |  |  |
| Quartile 1 (most with no diploma) | 138,079 (13.6) | 130,342 (13.4) | 6,997 (16.2) | 740 (17.5) |
| Quartile 2 | 211,328 (20.8) | 200,729 (20.7) | 9,555 (22.1) | 1,044 (24.7) |
| Quartile 3 | 270,983 (26.6) | 258,247 (26.6) | 11,573 (26.8) | 1,163 (27.5) |
| Quartile 4 (least with no diploma) | 259,298 (25.5) | 248,650 (25.6) | 9,790 (22.7) | 858 (20.3) |
| Missing/unknown | 138,531 (13.6) | 132,810 (13.7) | 5,291 (12.2) | 430 (10.2) |
| Cancer Program Type* |  |  |  |  |
| Community | 69,774 (7.1) | 66,069 (7.0) | 3,230 (8.0) | 475 (12.0) |
| Comprehensive Community | 413,606 (41.9) | 394,581 (41.9) | 17,287 (42.7) | 1,738 (43.8) |
| Academic/Research | 287,329 (29.1) | 274,538 (29.1) | 11,785 (29.1) | 1,006 (25.4) |
| Integrated Network | 215,850 (21.9) | 206,890 (22.0) | 8,215 (20.3) | 745 (18.8) |
| Histology |  |  |  |  |
| Ductal | 921,060 (90.5) | 878,742 (90.5) | 38,596 (89.3) | 3,722 (87.9) |
| Lobular | 88,381 (8.7) | 83,787 (8.6) | 4,134 (9.6) | 460 (10.9) |
| Other | 8,778 (0.9) | 8,249 (0.8) | 476 (1.1) | 53 (1.3) |
| Grade^#^ |  |  |  |  |
| Well Diff/Grade 1/ Low Nuclear Grade | 256,906 (25.2) | 251,234 (25.9) | 5,313 (12.3) | 359 (8.5) |
| Moderately Diff/Grade 2/Intermediate Nucl Gr | 451,446 (44.3) | 429,962 (44.3) | 19,753 (45.7) | 1,731 (40.9) |
| Poorly Diff/Grade 3/High Nuclear Gr/Undifferentiated | 251,547 (24.7) | 233,138 (24.0) | 16,460 (38.1) | 1,949 (46.0) |
| Grade Unknown/Not Available/Missing | 256,906 (25.2) | 251,234 (25.9) | 5,313 (12.3) | 359 (8.5) |
| Clinical Stage |  |  |  |  |
| 0 | 187,443 (18.4) | 187,394 (19.3) | *** | *** |
| 1 | 24,121 (2.4) | 24,037 (2.5) | 84 (0.2) | 0 |
| 1A | 579,069 (56.9) | 578,253 (59.6) | 804 (1.9) | 12 (0.3) |
| 1B | 43,032 (4.2) | 40,069 (4.1) | 2,939 (6.8) | 24 (0.6) |
| 2 | 3,880 (0.4) | *** | *** | 16 (0.4) |
| 2A | 142,591 (14.0) | 124,658 (12.8) | 17,620 (40.8) | 313 (7.4) |
| 2B | 24,916 (2.4) | 4,855 (0.5) | 19,975 (46.2) | 86 (2.0) |
| 3 | 163 (0.0) | *** | 81 (0.2) | *** |
| 3A | 4,042 (0.4) | 117 (0.0) | 326 (0.8) | 3,599 (85.0) |
| 3B | 362 (0.0) | 15 (0.0) | 318 (0.7) | 29 (0.7) |
| 3C | 82 (0.0) | 15 (0.0) | 23 (0.1) | 44 (1.0) |
| Unknown/Missing | 8,518 (0.8) | 8,136 (0.8) | 351 (0.8) | 31 (0.7) |
| ER Status |  |  |  |  |
| Negative | 126,373 (12.4) | 118,509 (12.2) | 7,022 (16.3) | 842 (19.9) |
| Positive | 884,220 (86.8) | 844,797 (87.0) | 36,049 (83.4) | 3,374 (79.7) |
| Missing/Unknown/Borderline | 7,626 (0.7) | 7,472 (0.8) | 135 (0.3) | 19 (0.4) |
| PR Status |  |  |  |  |
| Negative | 209,906 (20.6) | 197,757 (20.4) | 10,901 (25.2) | 1,248 (29.5) |
| Positive | 785,477 (77.1) | 750,388 (77.3) | 32,130 (74.4) | 2,959 (69.9) |
| Missing/Unknown/Borderline | 22,836 (2.2) | 22,633 (2.3) | 175 (0.4) | 28 (0.7) |
| HER2 Status |  |  |  |  |
| Negative | 777,403 (76.3) | 736,684 (75.9) | 37,288 (86.3) | 3,431 (81.0) |
| Positive | 63,713 (6.3) | 58,664 (6.0) | 4,432 (10.3) | 617 (14.6) |
| Missing/Unknown/Borderline | 177,103 (17.4) | 175,430 (18.1) | 1,486 (3.4) | 187 (4.4) |
| Phenotype |  |  |  |  |
| ER+/PR+, HER2-** | 705,243 (69.3) | 669,906 (69.0) | 32,462 (75.1) | 2,875 (67.9) |
| HER2+** | 63,427 (6.2) | 58,395 (6.0) | 4,416 (10.2) | 616 (14.5) |
| Triple Neg** | 70,832 (7.0) | 65,536 (6.8) | 4,751 (11.0) | 545 (12.9) |
| Missing//Unknown/Borderline | 28,271 (2.8) | 26,538 (2.7) | 1,538 (3.6) | 195 (4.6) |
| Tis ER+ or PR+ | 123,719 (12.2) | 123,690 (12.7) | *** | *** |
| Tis ER-/PR- | 19,844 (1.9) | 19,837 (2.0) | *** | *** |
| Tis ER and PR Missing | 4,983 (0.5) | 4,978 (0.5) | *** | *** |
| Tis ER- or PR-, Other Missing | 1,900 (0.2) | 1,898 (0.2) | *** | *** |
| cT Stage |  |  |  |  |
| Any Tis | 187,850 (18.4) | 187,734 (19.3) | *** | *** |
| cT1mi | 7,652 (0.8) | 7,594 (0.8) | *** | *** |
| cT1a | 58,399 (5.7) | 57,607 (5.9) | 736 (1.7) | 56 (1.3) |
| cT1b | 187,893 (18.5) | 185,255 (19.1) | 2,508 (5.8) | 130 (3.1) |
| cT1c | 280,982 (27.6) | 267,746 (27.6) | 12,369 (28.6) | 867 (20.5) |
| cT1 NOS | 106,725 (10.5) | 103,234 (10.6) | 3,301 (7.6) | 190 (4.5) |
| cT2 | 188,718 (18.5) | 161,608 (16.6) | 24,130 (55.8) | 2,980 (70.4) |
| cN Stage |  |  |  |  |
| cN0 | 187,850 (18.4) | 187,734 (19.3) | 105 (0.2) | 11 (0.3) |
| cN1 | 641,651 (63.0) | 621,436 (64.0) | 18,971 (43.9) | 1,244 (29.4) |
| cN2 | 188,718 (18.5) | 161,608 (16.6) | 24,130 (55.8) | 2,980 (70.4) |
| Surgery Type |  |  |  |  |
| Breast Conservation | 686,855 (67.5) | 666,884 (68.7) | 18,615 (43.1) | 1,356 (32.0) |
| Mastectomy | 331,364 (32.5) | 303,894 (31.3) | 24,591 (56.9) | 2,879 (68.0) |
| Nodal Procedures^∞^ **** |  |  |  |  |
| None/unknown | 87,369 (8.6) | 87,182 (9.0) | 167 (0.4) | 20 (0.5) |
| Performed, But Not Otherwise Specified | 147,752 (14.5) | 135,885 (14.0) | 10,450 (24.2) | 1,417 (33.5) |
| Sentinel Node Biopsy (or Excisional Biopsy) | 602,132 (59.1) | 594,249 (61.2) | 7,599 (17.6) | 284 (6.7) |
| ALND | 67,446 (6.6) | 50,018 (5.2) | 15,849 (36.7) | 1,579 (37.3) |
| Sentinel Node Biopsy & ALND Simultaneously | 103,923 (10.2) | 95,323 (9.8) | 7,788 (18.0) | 812 (19.2) |
| Sentinel Node Biopsy and Subsequent ALND | 9,597 (0.9) | 8,121 (0.8) | 1,353 (3.1) | 123 (2.9) |

*Cancer program type variable in the NCDB excludes patients<40. This variable includes a subset of 986,559 patients.

^#^Variables in different years that combines differentiation, grade, and nuclear grade are combined. Diff = differentiated, Gr = grade. Grade defined as clinical grade where available (2018+) and pathologic grade otherwise.

**ER = estrogen receptor-positive, PR = progesterone receptor-positive, HER2 = human epidermal growth factor 2-positive. Phenotype for invasive disease for these categories requires complete data for ER, PR and HER2.
***Cells <10 or those enabling calculation of those cells have been censored as per the requirements of the National Cancer Database.

^∞^ALND = Axillary lymph node dissection (any number of nodes)

****Unknown included with None as at least one Unknown cell was <10

**Supplementary Table 4. The adjusted probabilities of primary tumor upstaging by clinical T stage and by nodal upstaging for cN0 patients, stratified by ductal versus lobular histology, for each 30-day delay increment between diagnosis and surgery.** Results of 6 separate logistic regression models, two for each cT group and also two for cN0. The outcome is upstaging (yes/no), with delay from diagnosis to surgery included as a continuous variable (30-day intervals). Covariates included age, gender, race, Hispanicity, pathologic grade, histology, and phenotype. For cT1 and cT2, cN stage is included as a covariate. Some calculations may not add precisely due to rounding.

|  | **cT1** | | | | | | **cT2** | | | | | |
| --- | --- | --- | --- | --- | --- | --- | --- | --- | --- | --- | --- | --- |
|  | **Ductal** | | | **Lobular** | | | **Ductal** | | | **Lobular** | | |
|  | **Adjusted Probability of Upstaging, %** | | | **Adjusted Probability of Upstaging, %** | | | **Adjusted Probability of Upstaging, %** | | | **Adjusted Probability of Upstaging, %** | | |
| **Delay Interval** | **Est.** | **95%CI** | **Delay-Attributable** | **Est.** | **95%CI** | **Delay-Attributable** | **Est.** | **95%CI** | **Delay-Attributable** | **Est.** | **95%CI** | **Delay-Attributable** |
| Staging Inaccuracy^#^ | 10.1 | (9.8-10.4) | 0 | 20.9 | (20.2-21.6) | 0 | 3.3 | (3.1-3.4) | 0 | 13.8 | (12.9-14.6) | 0 |
| 30 days | 10.7 | (10.4-11.0) | 0.6 | 21.7 | (21.1-22.3) | 0.8 | 3.6 | (3.4-3.7) | 0.3 | 14.2 | (13.5-14.9) | 0.4 |
| 60 days | 11.9 | (11.6-12.2) | 1.8 | 23.4 | (22.7-24.1) | 2.5 | 4.3 | (4.1-4.5) | 1.1 | 15.1 | (14.3-15.8) | 1.3 |
| 90 days | 13.2 | (12.8-13.7) | 3.1 | 25.2 | (24.1-26.3) | 4.3 | 5.2 | (4.9-5.5) | 1.9 | 16.0 | (14.7-17.2) | 2.2 |
| 120 days | 14.7 | (14.1-15.4) | 4.6 | 27.1 | (25.4-28.8) | 6.2 | 6.3 | (5.7-6.8) | 3.0 | 16.9 | (15.0-18.9) | 3.2 |
| 150 days | 16.3 | (15.4-17.2) | 6.2 | 29.1 | (26.7-31.4) | 8.2 | 7.5 | (6.6-8.4) | 4.2 | 17.9 | (15.2-20.7) | 4.2 |
| 180 days | 18.0 | (16.8-19.2) | 7.9 | 31.1 | (28.1-34.2) | 10.2 | 9.0 | (7.7-10.2) | 5.7 | 19.0 | (15.3-22.7) | 5.2 |
|  |  |  |  |  |  |  |  |  |  |  |  |  |
| **Odds Ratio for Delay (difference of 30 days):** | | |  |  |  |  |  |  |  |  |  |  |
|  | **cT1, Ductal** | | | **cT1, Lobular** | | | **cT2, Ductal** | | | **cT2, Lobular** | | |
|  | **OR** | **95% CI** |  | **OR** | **95% CI** |  | **OR** | **95% CI** |  | **OR** | **95% CI** |  |
|  | 1.13 | 1.11-1.15 | p<0.0001 | 1.10 | 1.07-1.14 | p<0.0001 | 1.22 | 1.18-1.26 | p<0.0001 | 1.07 | 1.02-1.13 | p=0.009 |

|  | **cN0 (cTis excluded)** | | | | | |
| --- | --- | --- | --- | --- | --- | --- |
|  | **Ductal** | | | **Lobular** | | |
|  | **Adjusted Probability of Nodal Upstaging, %** | | | **Adjusted Probability of Nodal Upstaging, %** | | |
| **Delay Interval** | **Est.** | **95%CI** | **Delay-Attributable** | **Est.** | **95%CI** | **Delay-Attributable** |
| Staging Inaccuracy^#^ | 16.2 | (15.9-16.4) | 0 | 20.2 | (19.7-20.7) | 0 |
| 30 days | 16.6 | (16.4-16.8) | 0.4 | 20.4 | (20.0-20.8) | 0.2 |
| 60 days | 17.5 | (17.2-17.7) | 1.3 | 20.8 | (20.3-21.2) | 0.5 |
| 90 days | 18.4 | (18.0-18.7) | 2.2 | 21.1 | (20.4-21.8) | 0.9 |
| 120 days | 19.3 | (18.8-19.8) | 3.2 | 21.5 | (20.4-22.6) | 1.2 |
| 150 days | 20.3 | (19.6-21.0) | 4.2 | 21.8 | (20.3-23.4) | 1.6 |
| 180 days | 21.3 | (20.5-22.2) | 5.2 | 22.2 | (20.3-24.2) | 2.0 |
|  |  |  |  |  |  |  |
| **Odds Ratio for Delay (difference of 30 days):** | | |  |  |  |  |
|  | **cN0, Ductal** | | | **cN0, Lobular** | | |
|  | **OR** | **95% CI** |  | **OR** | **95% CI** |  |
|  | 1.07 | 1.06-1.08 | p<0.0001 | 1.02 | 1.00-1.05 | 0.084 |

**Supplementary Table 5. The adjusted probabilities of primary tumor upstaging by phenotype, stratified by ductal versus lobular histology, for each 30-day delay increment between diagnosis and surgery.** Results of 6 separate logistic regression models, 2 for each invasive phenotype group (hormone receptor-positive [HR+], human epidermal growth factor 2-positive [HER2+] and triple negative [TN]). Phenotypes that were not discernible due to missing data were excluded. The outcome is upstaging (yes/no), with the delay between diagnosis and surgery included as a continuous variable (30-day intervals). Covariates included age, gender, race, Hispanicity, grade, histology, cT stage (cT1 or cT2) and cN stage. Clinical grade was used where available (2018 onward) with pathologic grade used otherwise. Insignificant delay-attributable upstaging for lobular tumors of HER2+ and TN phenotypes appear to be due to small sample sizes (*n*=1,845 and *n*=717, respectively). Some calculations may not add precisely due to rounding.

|  | **HR+** | | | | | | **HER2+** | | | | | |
| --- | --- | --- | --- | --- | --- | --- | --- | --- | --- | --- | --- | --- |
|  | **Ductal** | | | **Lobular** | | | **Ductal** | | | **Lobular** | | |
|  | **Adjusted Probability of Upstaging, %** | | | **Adjusted Probability of Upstaging, %** | | | **Adjusted Probability of Upstaging, %** | | | **Adjusted Probability of Upstaging, %** | | |
| **Delay Interval** | **Est.** | **95%CI** | **Delay-Attributable** | **Est.** | **95%CI** | **Delay-Attributable** | **Est.** | **95%CI** | **Delay-Attributable** | **Est.** | **95%CI** | **Delay-Attributable** |
| Staging Inaccuracy | 8.4 | (8.2-8.7) | 0 | 19.1 | (18.4-19.7) | 0 | 9.7 | (9.2-10.1) | 0 | 14.8 | (12.6-17.1) | 0 |
| 30 days | 8.9 | (8.7-9.2) | 0.5 | 19.8 | (19.3-20.3) | 0.7 | 10.0 | (9.7-10.4) | 0.3 | 15.7 | (14.0-17.5) | 0.9 |
| 60 days | 10.1 | (9.8-10.3) | 1.6 | 21.3 | (20.7-21.9) | 2.3 | 10.7 | (10.2-11.2) | 1.0 | 17.7 | (15.2-20.1) | 2.8 |
| 90 days | 11.3 | (10.9-11.7) | 2.9 | 22.9 | (22.0-23.9) | 3.9 | 11.5 | (10.6-12.3) | 1.8 | 19.8 | (15.0-24.5) | 4.9 |
| 120 days | 12.7 | (12.1-13.2) | 4.2 | 24.6 | (23.2-26.0) | 5.6 | 12.2 | (10.9-13.6) | 2.5 | 22.1 | (14.3-29.8) | 7.2 |
| 150 days | 14.2 | (13.4-15.0) | 5.7 | 26.4 | (24.4-28.3) | 7.3 | 13.1 | (11.2-14.9) | 3.4 | 24.5 | (13.4-35.7) | 9.7 |
| 180 days | 15.8 | (14.7-16.9) | 7.4 | 28.2 | (25.7-30.8) | 9.2 | 13.9 | (11.5-16.4) | 4.2 | 27.2 | (12.2-42.1) | 12.3 |
|  |  |  |  |  |  |  |  |  |  |  |  |  |
| **Odds Ratio for Delay (difference of 30 days):** | | |  |  |  |  |  |  |  |  |  |  |
|  | **HR+, Ductal** | | | **HR+, Lobular** | | | **HER2+, Ductal** | | | **HER2+, Lobular** | | |
|  | **OR** | **95% CI** |  | **OR** | **95% CI** |  | **OR** | **95% CI** |  | **OR** | **95% CI** |  |
|  | 1.15 | 1.13-1.17 | p<0.0001 | 1.10 | 1.07-1.13 | p<0.0001 | 1.08 | 1.03-1.13 | p=0.0006 | 1.15 | 0.98-1.36 | p=0.09 |
|  |  |  |  |  |  |  |  |  |  |  |  |  |
|  |  |  |  |  |  |  |  |  |  |  |  |  |
|  | **Triple Negative** | | | | | |  |  |  |  |  |  |
|  | **Ductal** | | | **Lobular** | | |  |  |  |  |  |  |
|  | **Adjusted Probability of Nodal Upstaging, %** | | | **Adjusted Probability of Nodal Upstaging, %** | | |  |  |  |  |  |  |
| **Delay Interval** | **Est.** | **95%CI** | **Delay-Attributable** | **Est.** | **95%CI** | **Delay-Attributable** |  |  |  |  |  |  |
| Staging Inaccuracy | 10.0 | (9.6-10.4) | 0 | 20.3 | (16.2-24.4) | 0 |  |  |  |  |  |  |
| 30 days | 10.9 | (10.5-11.3) | 0.9 | 19.8 | (16.8-22.8) | -0.5 |  |  |  |  |  |  |
| 60 days | 12.8 | (12.3-13.3) | 2.8 | 18.8 | (14.6-22.9) | -1.6 |  |  |  |  |  |  |
| 90 days | 15.0 | (14.1-15.9) | 5.0 | 17.8 | (10.6-24.9) | -2.5 |  |  |  |  |  |  |
| 120 days | 17.4 | (16.0-18.9) | 7.4 | 16.8 | (6.6-27.1) | -3.5 |  |  |  |  |  |  |
| 150 days | 20.2 | (18.0-22.4) | 10.2 | 15.9 | (2.8-29.1) | -4.4 |  |  |  |  |  |  |
| 180 days | 23.2 | (20.2-26.2) | 13.2 | 15.1 | (-0.7-30.8) | -5.3 |  |  |  |  |  |  |
|  |  |  |  |  |  |  |  |  |  |  |  |  |
| **Odds Ratio for Delay (difference of 30 days):** | | |  |  |  |  |  |  |  |  |  |  |
|  | **TN, Ductal** | | | **TN, Lobular** | | |  |  |  |  |  |  |
|  | **OR** | **95% CI** |  | **OR** | **95% CI** |  |  |  |  |  |  |  |
|  | 1.21 | 1.17-1.26 | p<0.0001 | 0.93 | 0.72-1.21 | p=0.61 |  |  |  |  |  |  |

Note: cT=Tis excluded, as phenotype is for invasive primary tumors. Age is a linear continuous variable (in the logistic model), gender defined as male vs female, and race was categorized as White, Black, Asian, and other/unknown. Grade was categorized into four groups: grade 1 or well differentiated, grade 2 or moderately differentiated, grade 3 or poorly differentiated or undifferentiated, and grade unknown or missing. Histology was grouped into ductal, lobular, and other/unknown.

^#^Inaccuracy refers to the baseline understaging rate that occurs without significant delay. This is calculated by determining the rate of upstaging for patients having times between diagnosis and surgery ≤15 days, assuming negligible growth within that time period. The delay-attributable portion is calculated by subtracting this baseline inaccuracy from the upstaging rate associated with significant delay, to achieve the upstaging rate attributable due to delay.
