# Supplementary material for: Breast Cancer Upstaging Risk and In Vivo Tumor Growth Rates Associated with Preoperative Delays
Source: Ann Surg Oncol. 2025 Jul 23;32(12):8789–97. doi: 10.1245/s10434-025-17867-9 (PMC12534249; doi:10.1245/s10434-025-17867-9)

**SUPPLEMENTARY FIGURES (Bleicher, *Ann Surg Oncol* 2025)**

**Supplementary Figure 1. Histogram for interval (days) between diagnosis and surgery for 1,018,219 patients.**

Distribution frequency shown below.

Descriptive statistics (days):

Mean: 38.5 ± 22.2 (standard deviation).

Range: 1-180.

Median: 34.0.

Percentiles (10^th^, 25^th^, 75^th^, 90^th^): 15.0, 23.0, 49.0, 66.0.


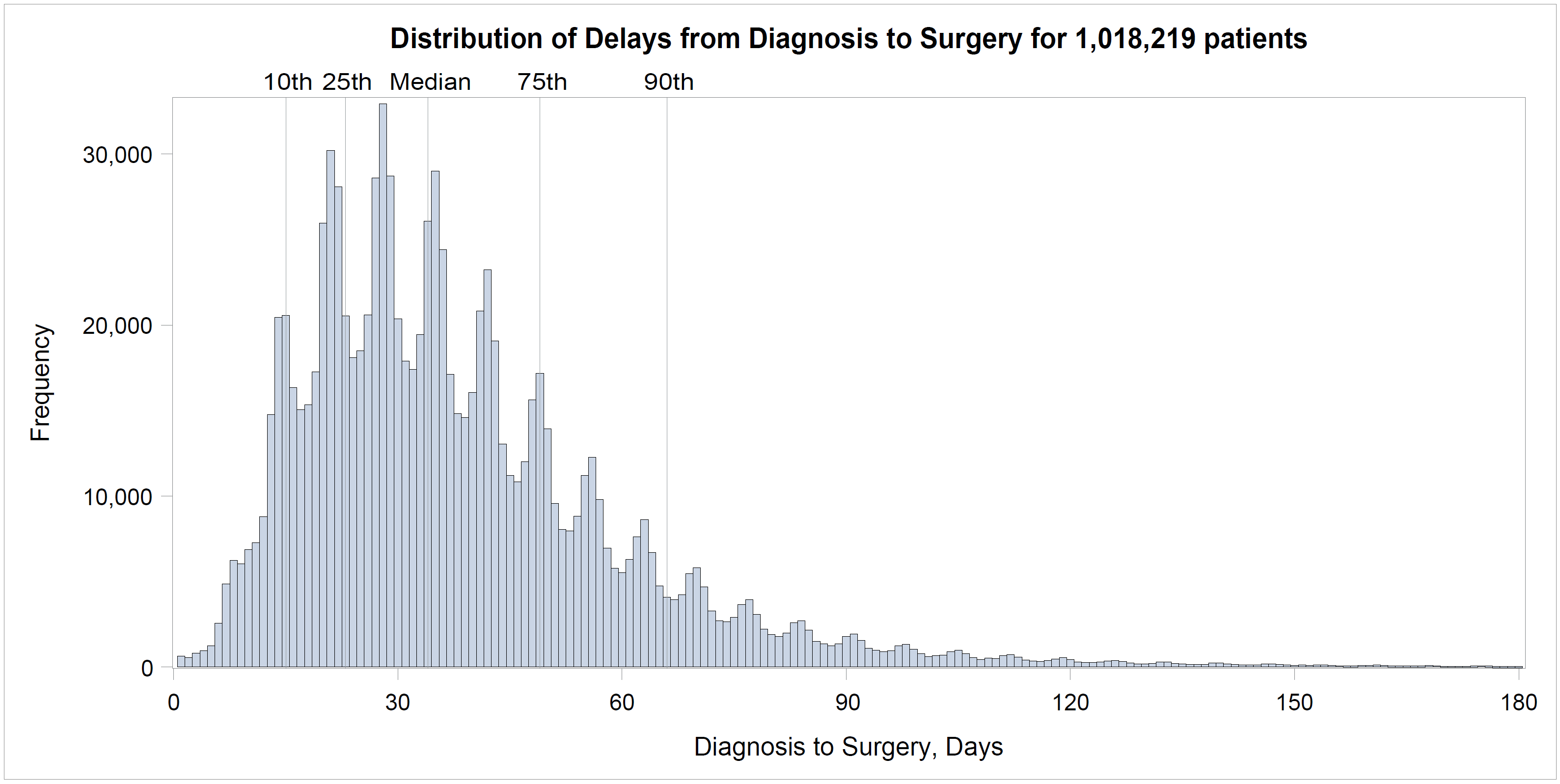


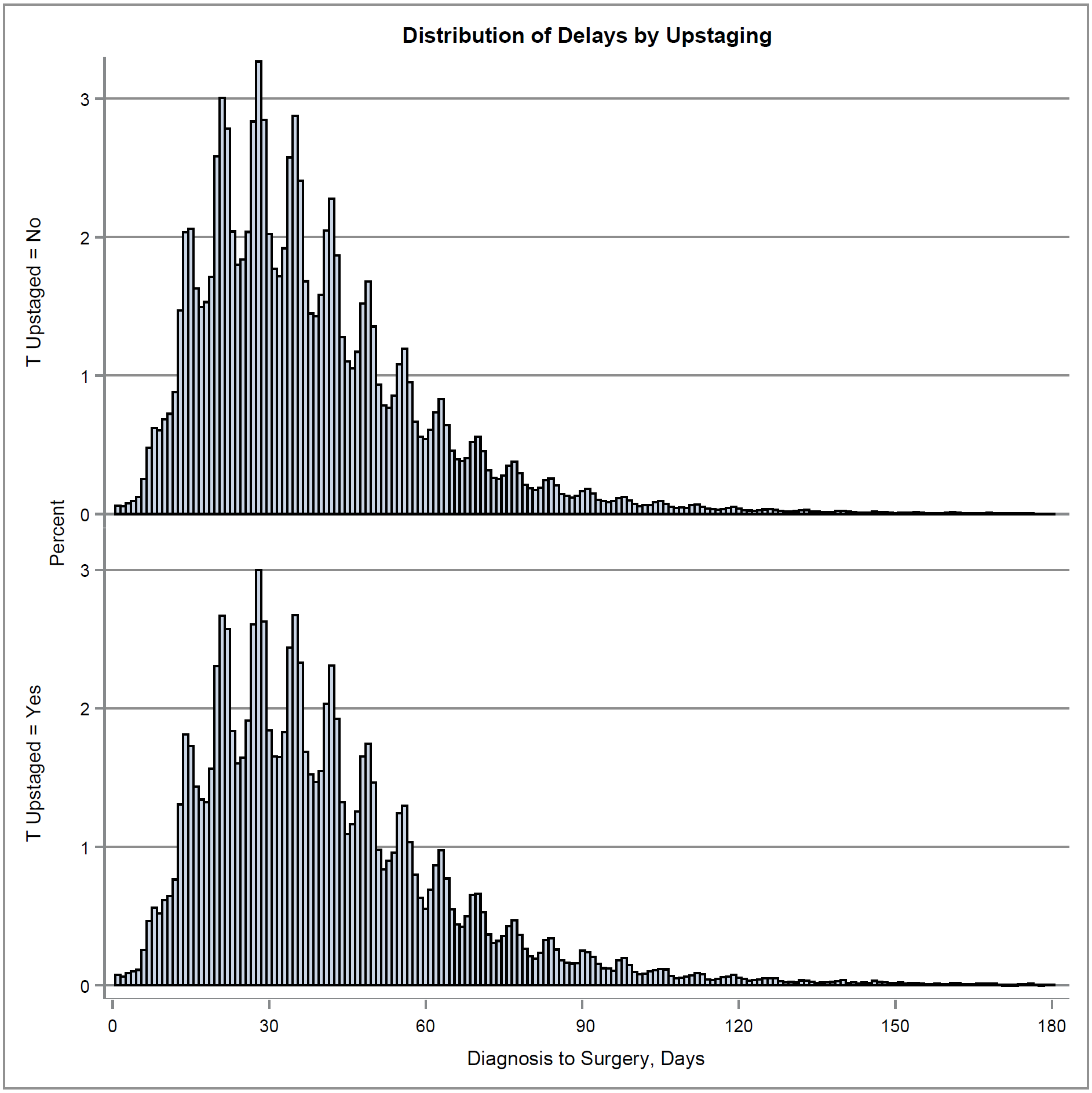
**Supplementary Figure 2. Histogram of interval (days) between diagnosis and surgery, stratified by whether the patient upstaged or did not upstage in that interval/ for 1,018,219 patients.**

**Did not upstage.**

Mean: 38.2 ± 22.0

Range: 1-180.

Median: 34.0.

Percentiles (10^th^, 25^th^, 75^th^, 90^th^):

15.0, 23.0, 48.0, 66.0.

**Did upstage.**

Mean: 40.8 ± 23.8.

Range: 1-180.

Median: 36.0.

Percentiles (10^th^, 25^th^, 75^th^, 90^th^):

16.0, 24.0, 51.0, 71.0.

**Supplementary Figure 3. Percentage of patients upstaged 1 level or >1 level, enumerated by stage, phenotype, and for cN0 patients.**


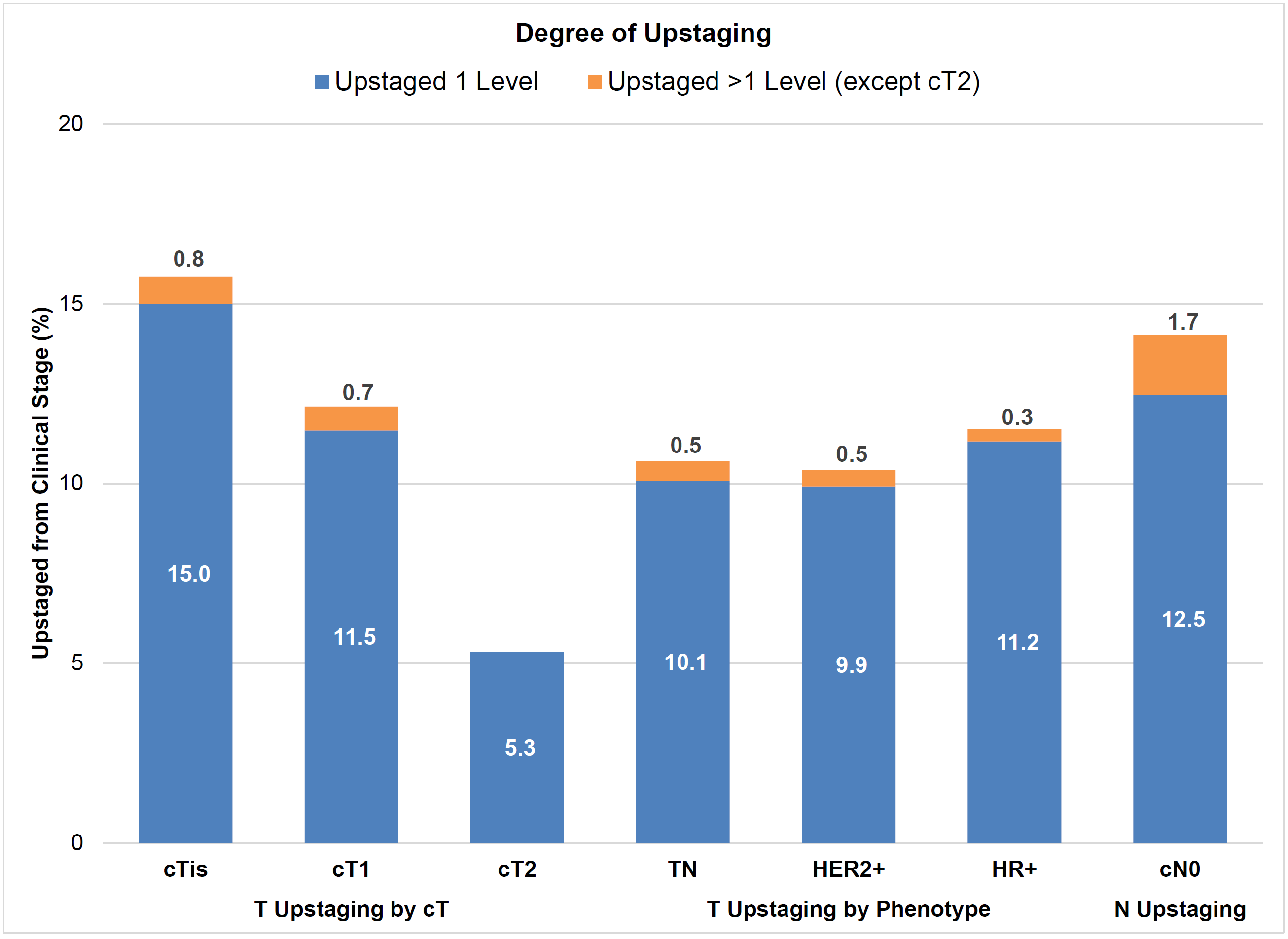


**Supplementary Figure 4. Adjusted probabilities of upstaging.** (a) upstaging of the primary tumors for cTis, cT1 and cT2 tumors, (b) upstaging of the primary tumors for triple negative, hormone receptor-positive, and HER2-positive, and (c) upstaging of the nodes from cN0 to node-positive. TN = triple negative, HR+ = hormone receptor positive (estrogen receptor-positive and/or progesterone receptor-positive), HER2+ = human epidermal growth factor-positive.

**
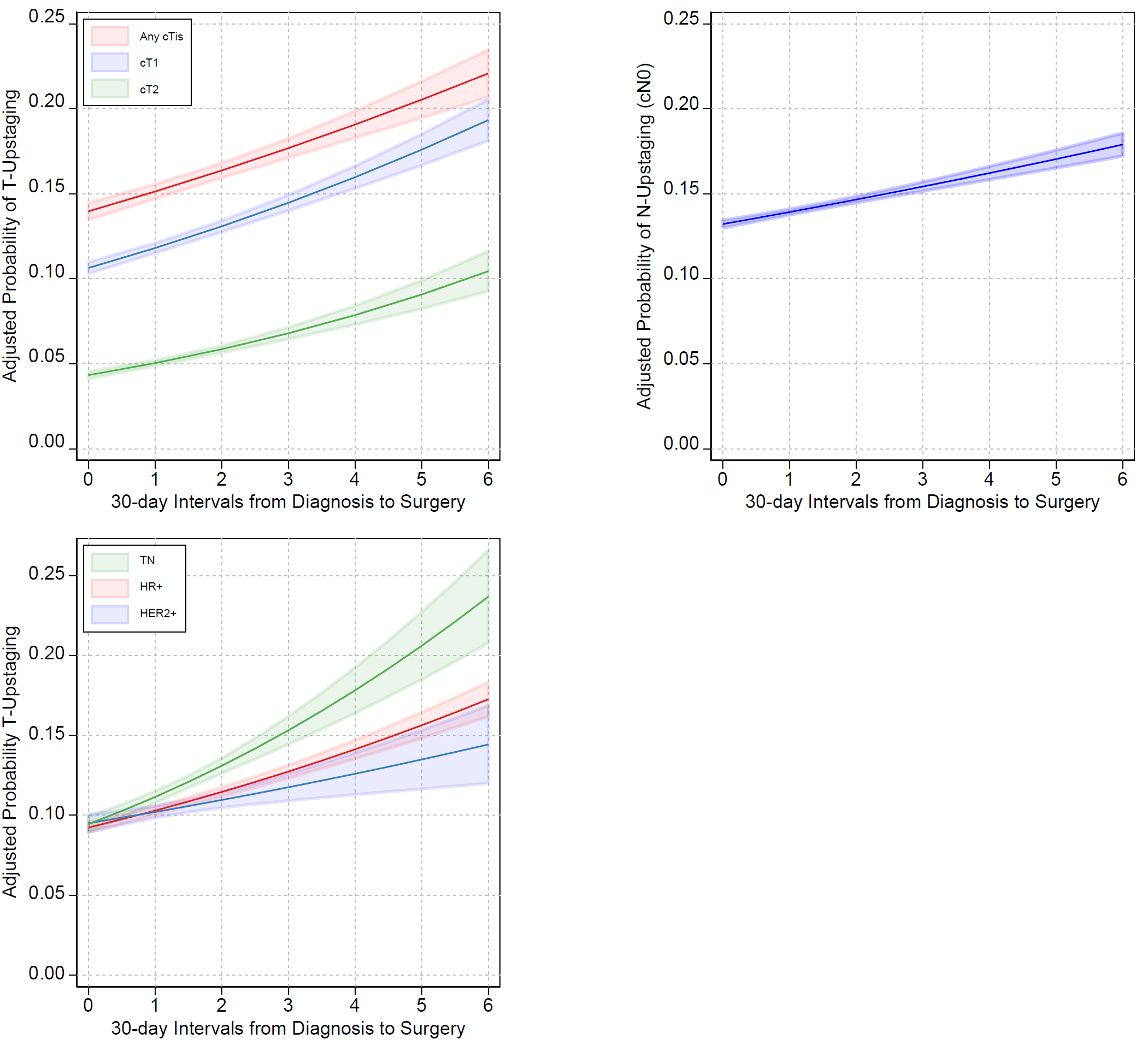
**

**Supplementary Figure 5. Concatenated regression curves demonstrating the rate of growth by T stage.** The slopes of growth rates from regression analysis *in vivo*, as depicted in **Figure 2**, are shown in in consecutive fashion demonstrating the likely increasing rate of growth by tumor size/substage as tumors enlarge, along with extrapolated time points at which tumors would theoretically reach the next stage, consistent with prior theories on increasing tumor growth rates with size. Solid lines represent 95% of the data in the regression with dotted lines representing extrapolation to the next higher size/stage.


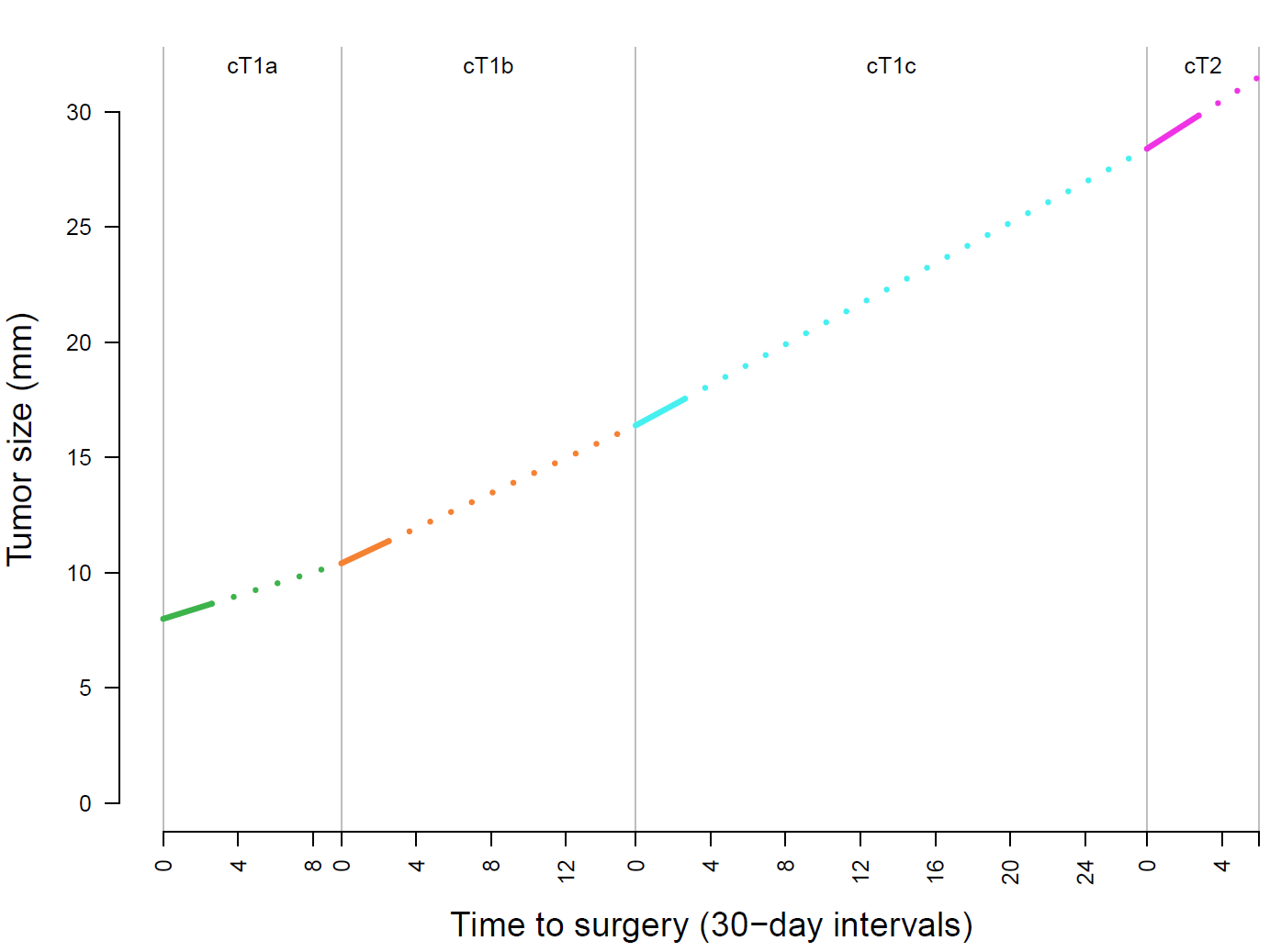

Supplement: Supplementary file 3 — Supplementary file3 (DOCX 677 KB) [file 10434_2025_17867_MOESM3_ESM.docx]
